# The SAVED domain of the type III CRISPR protease CalpL is a ring nuclease

**DOI:** 10.1093/nar/gkae676

**Published:** 2024-08-21

**Authors:** Sophie C Binder, Niels Schneberger, Maximilian Schmitz, Marianne Engeser, Matthias Geyer, Christophe Rouillon, Gregor Hagelueken

**Affiliations:** Institute of Structural Biology, Venusberg-Campus 1, University of Bonn, Bonn, Germany; Institute of Structural Biology, Venusberg-Campus 1, University of Bonn, Bonn, Germany; Institute of Structural Biology, Venusberg-Campus 1, University of Bonn, Bonn, Germany; Kekulé Institute of Organic Chemistry and Biochemistry, University of Bonn, Bonn, Germany; Institute of Structural Biology, Venusberg-Campus 1, University of Bonn, Bonn, Germany; Institut Pasteur, Université Paris Cité, Synthetic Biology, 75015 Paris, France; Institute of Structural Biology, Venusberg-Campus 1, University of Bonn, Bonn, Germany

## Abstract

Prokaryotic CRISPR-Cas immune systems detect and cleave foreign nucleic acids. In type III CRISPR-Cas systems, the Cas10 subunit of the activated recognition complex synthesizes cyclic oligoadenylates (cOAs), second messengers that activate downstream ancillary effector proteins. Once the viral attack has been weathered, elimination of extant cOA is essential to limit the antiviral response and to allow cellular recovery. Various families of ring nucleases have been identified, specializing in the degradation of cOAs either as standalone enzymes or as domains of effector proteins. Here we describe the ring nuclease activity inherent in the SAVED domain of the cA_4_-activated CRISPR Lon protease CalpL. We characterize the kinetics of cA_4_ cleavage and identify key catalytic residues. We demonstrate that cA_4_-induced oligomerization of CalpL is essential not only for activation of the protease, but is also required for nuclease activity. Further, the nuclease activity of CalpL poses a limitation to the protease reaction, indicating a mechanism for regulation of the CalpL/T/S signaling cascade. This work is the first demonstration of a catalytic SAVED domain and gives new insights into the dynamics of transcriptional adaption in CRISPR defense systems.

## Graphical Abstract

**Graphical Abstract:**
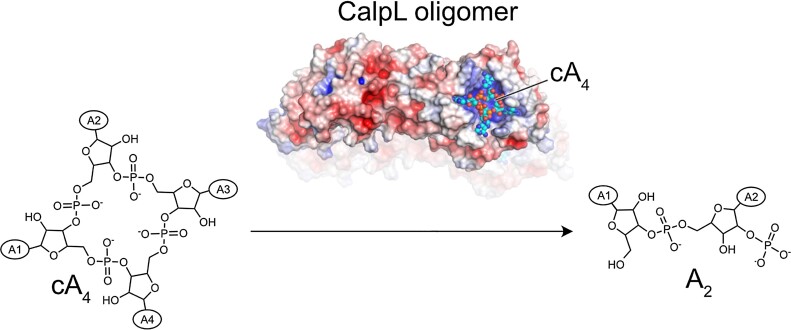

## Introduction

Bacteria are constantly threatened by the attack of foreign genetic elements, such as phages, and have evolved immune systems of varying complexity, such as restriction enzymes, toxin-antitoxin systems, CBASS, CRISPR and many others as a countermeasure (1,2). In CRISPR systems (3), a memory of previous phage attacks is stored in the form of short snippets of phage DNA in the CRISPR array of the bacterial chromosome (4). Those snippets are transcribed into short RNAs that are incorporated into recognition complexes sensing the presence of complementary foreign DNA or viral transcripts in the cell (5). If an attack is detected, the response can range from simple cleavage of the foreign DNA in the well-known Type II CRISPR systems (Cas9) to complex multi-pronged responses in Type III CRISPR systems (6–13). In the latter, the Cas10 subunit of the recognition complex leads to the synthesis of cyclic oligoadenylates (cOAs), which are recognized by the CARF- or SAVED domains of effector proteins (14–16). These can have a variety of biological functions such as DNAses, RNAses, nickases, transcription factors (13), or proteases as shown for CalpL (CRISPR-associated Lon protease), Craspase (CRISPR RNA-guided Caspase), the TPR-CHAT protease or the recently discovered SAVED-CHAT protein (16–19).

CalpL is part of the tripartite CalpL/T/S complex found in the thermophilic bacterium *Sulfurihydrogenibium* sp. YO3AOP1. The complex is formed by the protease CalpL, the anti-σ-factor CalpT and the extracytoplasmic function (ECF) σ-factor CalpS. Under normal conditions, the σ-factor is inhibited by CalpT. Under viral attack, cA_4_ is produced and the C-terminal SAVED domain of CalpL binds the second messenger with nanomolar affinity. This leads to oligomerization of CalpL and activation of the N-terminal Lon protease domain, which cleaves the 33 kDa anti- σ-factor CalpT, resulting in release of the 23 kDa CalpT_23_ fragment bound to the σ-factor CalpS. In analogy to other anti-σ-factor/σ-factor complexes, degradation by the ClpX/P-degron system is thought to release the sigma factor and allow the cell to adapt to a viral attack (16,20).

Both bacteria and viruses have evolved mechanisms to either regulate or inhibit the antiviral response by degrading the cOA second messengers. This reaction is catalyzed by ring nucleases, which can be found as dedicated enzymes such as the archaeal host protein *Sso*2081 and the viral AcrIII-1 protein or as an intrinsic activity of the CARF domain effector proteins such as *Sis*0811, *Sis*0455 or the HEPN ribonuclease TTHB144 (13,21–25).

**Figure 1.**
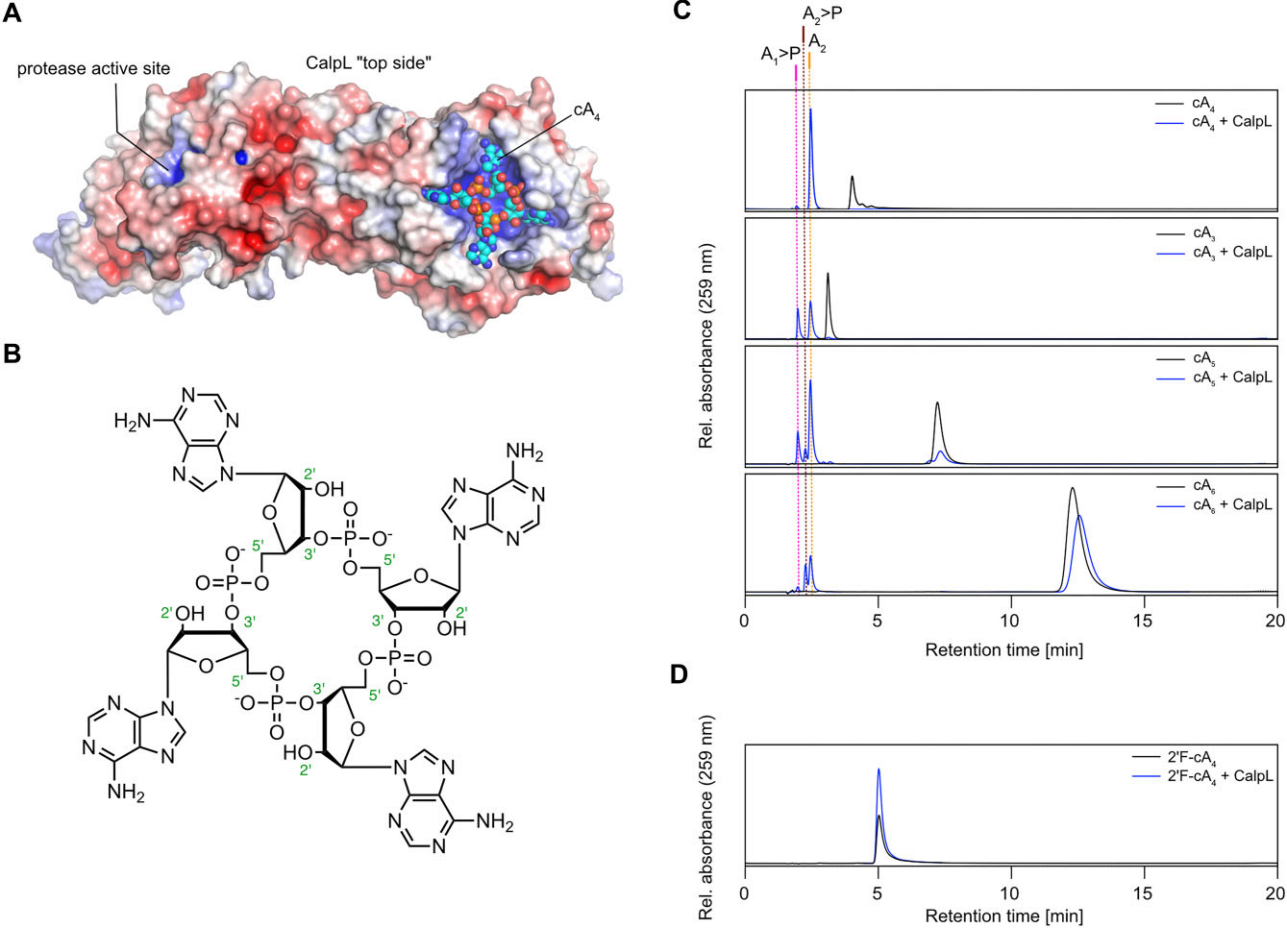
CalpL is a ring nuclease. (**A**) A surface representation of a CalpL monomer with the electrostatic surface potential mapped onto the structure (red negative, blue positive). The cA_4_ molecule bound to the SAVED domain is shown in ball-and-sticks representation (PDB-ID: 8b0r, (16)). (**B**) Structural formula of cA_4_. The numbering of selected atoms is given in green. (**C**, **D**) HPLC traces recorded at 259 nm showing the result of incubating 30 μM of different cOAs with (blue lines) and without (black lines) 3 μM CalpL for 120 min at 60°C. The vertical lines mark reaction intermediates and products that were identified by comparison with standard runs and/or mass spectrometry. HPLC traces are representative of at least three replicates.

Here, we show that the SAVED domain of the CalpL protease (Figure 1A) has ring nuclease activity and that the 2′-OH group of cA_4_ is very likely the nucleophile in the ring nuclease reaction (Figure 1B). We analyze the reaction by time-resolved HPLC/MS and study the influence of the nuclease activity on the oligomerization of CalpL and on its protease activity.

## Materials and methods

### Generation of CalpL expression vectors

All CalpL constructs were expressed from a pET11a plasmid containing the codon-optimized sequence with an N-terminal 10xHis-TEV tag. Point mutations were introduced into plasmids by primer-directed mutagenesis. The non-mutated parental plasmid was digested by the methylation-sensitive restriction endonuclease *Dpn*I. After restriction digestion, 10 μl of the reaction mixture was used for transformation of NEB DH5α cells.

### Expression and purification of CalpL

Recombinant His10-CalpL was expressed in *Escherichia coli* BL21 (DE3) bacterial cells. *E. coli* cells were grown in LB medium containing the appropriate antibiotic at 37°C for 16 h (pre-culture). The following day, larger volumes of LB medium were inoculated with the pre-culture and adjusted to an optical density (OD_600_) of 0.1. Cultures were grown at 37°C to an OD_600_ of 0.8 and protein expression was induced by addition of IPTG to a final concentration of 0.4 mM. Proteins were expressed at 20°C for 16h. Bacteria were harvested by centrifugation at 4 000 × *g* for 25 min. Cell pellets were snap-frozen in liquid nitrogen and stored at –20°C or subjected to immediate cell lysis.

Cell pellets were resuspended in lysis buffer (20 mM Tris–HCl pH 8.0, 50 mM NaCl) and lysed by sonication. Cell debris was sedimented by centrifugation at 25 000 × *g* for 45 min at 10°C. The lysate was filtered through a membrane filter with a 0.8 μm pore size and subjected to affinity chromatography.

For affinity chromatography, Ni^2+^-NTA resin was equilibrated with lysis buffer and incubated with lysate for 2 h at 4°C on a rolling incubator. The resin was then transferred to a gravity column and washed extensively with wash buffer (20 mM Tris–HCl pH 8.0, 50 mM NaCl, 20 mM imidazole) and eluted with elution buffer (20 mM Tris–HCl pH 8.0, 50 mM NaCl, 500 mM imidazole). The elution fraction was dialyzed against lysis buffer for 16 h at 4°C and subjected to further purification by anion exchange chromatography.

Anion exchange chromatography was performed using a HiTrap Q High Performance quaternary ammonium anion exchange column (5 ml, Cytiva) equilibrated in binding buffer (20 mM Tris–HCl pH 8.0) using an ÄKTA FPLC system (Cytiva). The column was washed extensively with binding buffer and proteins were eluted by increasing the concentration of NaCl from 0 mM to 1 M over 12.5 column volumes (CV). Fractions containing large amounts of CalpL were pooled and digested with TEV protease (1:25 w/w) for 16 h at 4°C for removal of the 10xHis-tag.

Following protease digest, CalpL was further purified by size exclusion chromatography (SEC) on a HiLoad 16/600 Superdex 200 pg column (Cytiva) equilibrated with lysis buffer and reverse Ni^2+^-NTA purification for removal of residual tag, non-cleaved protein and TEV protease.

### Expression and purification of CalpT

CalpT was expressed from a pBAD plasmid containing the codon-optimized sequence with an N-terminal 6xHis-TEV tag. Recombinant 6xHis-CalpT was expressed in *Escherichia coli* BL21 (DE3) bacterial cells. *E. coli* cells were grown in LB medium containing the appropriate antibiotic at 37°C for 16 h (pre-culture). The following day, larger volumes of LB medium were inoculated with the pre-culture and adjusted to an optical density (OD_600_) of 0.1. Cultures were grown at 37°C to an OD_600_ of 0.8 and protein expression was induced by addition of 0.5 g/l l(+)-arabinose. Proteins were expressed at 30°C for 5 h. Bacteria were harvested by centrifugation at 4 000 × *g* for 25 min. Cell pellets were snap-frozen in liquid nitrogen and stored at –20°C or subjected to immediate cell lysis.

Cell pellets were resuspended in lysis buffer (25 mM Tris–HCl pH 8.0, 500 mM NaCl, 1 mM DTT, 10% glycerol) and lysed by sonication. Cell debris was sedimented by centrifugation at 25 000 × *g* for 45 min at 20°C. The lysate was filtered through a membrane filter with a 0.8 μm pore size and subjected to affinity chromatography.

For affinity chromatography, Ni^2+^-NTA resin was equilibrated with lysis buffer and incubated with lysate for 2 h at room temperature on a rolling incubator. The resin was then transferred to a gravity column and washed extensively with wash buffer (25 mM Tris–HCl pH 8.0, 500 mM NaCl, 1 mM DTT, 10% glycerol, 40 mM imidazole) and eluted with elution buffer (25 mM Tris–HCl pH 8.0, 500 mM NaCl, 1 mM DTT, 10% glycerol, 1 M imidazole). The elution fraction was dialyzed against binding buffer (25 mM Tris–HCl pH 8.0, 1 mM DTT, 10% glycerol) for 16 h at room temperature and subjected to further purification by Heparin chromatography.

Heparin chromatography was performed on a HiPrep Heparin Fast Flow 16/60 column (Cytiva) equilibrated in binding buffer using an ÄKTA FPLC system (Cytiva). The column was washed extensively with binding buffer and proteins were eluted by increasing the concentration of NaCl from 0 to 500 mM over 3.6 CVs. Fractions containing large amounts of 6xHis-CalpT were pooled and further purified by SEC on a HiLoad 16/600 Superdex75 pg column (Cytiva) equilibrated with 25 mM Tris–HCl, 500 mM NaCl, 1 mM DTT, 10% glycerol.

### Nuclease assay and RP-HPLC analyses

For testing the nuclease activity, CalpL was incubated with 10-fold molar excess of the respective cOA (Biolog) in 20 mM Tris–HCl, 50 mM NaCl at either 37°C (time-course measurement) or 60°C (endpoint measurement) and analyzed by reversed-phase high-performance liquid chromatography (RP-HPLC) using the Infinity II HPLC system (Agilent). Nuclease reaction products were separated at a flow-rate of 1 ml min^−1^ on a Chromolith Performance RP-18e 100 × 46 mm column (Merck) equipped with a guard cartridge. The eluent was composed of 30 mM K_2_HPO_4_, 70 mM KH_2_PO_4_, 10 mM tetrabutylammonium bromide, 13% acetonitrile (*w/w*). RNA species were collected manually and subjected to mass spectrometry for identification.

For calculation of rate constants *k*, the fraction of cA_4_ cut was determined by integrating the peak area at 259 nm for the intervals of *t* = 5 s to *t* = 600 s. Values were normalized to *t* = 5 s. For the CalpL variants H345A, H474A and wildtype, inversed values were fitted by a one phase decay model (*Y* = (*Y*0 – Plateau) × exp(–*K*×*X*) + Plateau) to the time points intervals of *t* = 5 s to *t* = 600 s using DataGraph 5.3. Due to the slow progression of the nuclease reaction catalyzed by the CalpL variants H392A, R361A and R361E, no adequate fit was applicable.

### Generation of HPLC standards

For generation of A_4_>P, A_3_>P and A_2_>P as HPLC standards similar to (26), 30 μM of MazF substrate (for A_4_>P: 5′-*aaaa*acacugaaccug-3′; for A_3_>P: 5′-*aaa*acacugaaccug-3′ for A_2_>P: 5′-*aa*acacugaaccug-3′) were incubated with 10 U recombinant MazF (Takara Bio) in 20 mM Tris pH 8.0, 50 mM NaCl and incubated for 60 min @ 37°C. Linear adenylate standards were ordered containing a 3′phosphate modification. MazF substrates and linear oligoadenylates were supplied by Metabion (Germany).

### Mass spectrometry analyses

Samples manually collected after HPLC separation were subjected to HPLC–MS analysis with a Q/TOF mass spectrometer (Bruker micrOTOF-Q) equipped with an electrospray ion source and a HPLC system (Agilent 1200 series) using a RP 150 × 2 mm C18 column (Knauer Eurospher II 100–5C18). The flow rate was set to 0.25 ml min^−1^ with a linear gradient starting from a 95:5 mixture of 25 mM aqueous ammonium acetate and acetonitrile ramping to a 30:70 ratio after 15 minutes. The column was rinsed with 5:95 NH_4_OAc:acetonitrile after every run. RNA samples were detected via UV absorption at 254 nm and ESI spectra measured in negative mode.

### Protease assay

To monitor protease activity over time, CalpL and 6xHis-CalpT were combined at 3 μM each in 20 mM Tris–HCl pH 8.0, 50 mM NaCl, and incubated at 37°C for 30 min. For activation of the protease, cA_4_ was added to a final concentration of 3 μM, and the reaction proceeded at 37°C for the indicated time periods. For endpoint analysis of protease reactions, the same protocol was followed but with a 30-min incubation at 60°C. After addition of cyclic or linear adenylate species, the reactions were incubated for further 60 minutes at 60°C. Reactions were stopped by addition of reducing SDS loading buffer and heating for 10 min at 94°C. The samples were loaded on a 15% SDS polyacrylamide gel, run at 40 mA/gel for 60 min and analyzed by Coomassie staining.

Cleavage of 6xHis-CalpT was assessed by quantifying CalpT bands on Coomassie-stained SDS-PAGE gels and normalizing to a non-cleaved 6xHis-CalpT control sample using the Image Lab 6.1 Software (BioRad). Inverse values were calculated for plotting. Fits were calculated from all data points ranging from 5 sec to 720 min (5 s, 15 s, 30 s, 90 s, 5 min, 10 min, 30 min, 60 min, 120 min, 240 min, 720 min). Due to an inadequate integration of CalpT band intensities for the CalpL R361E variant, quantification was done by integrating band intensities of CalpT_23_ and normalization to the amount of CalpT_23_ at *t* = 240 min. Rate constants *k* were calculated by fitting a one phase decay model (*Y* = (*Y*0 – Plateau) × exp(–*K*× *X*) + Plateau) to the time points intervals of *t* = 5 s to *t* = 720 min using DataGraph 5.3.

### Dynamic light scattering

To investigate cA_4_ induced oligomerization, the hydrodynamic radii of CalpL in the presence or absence of cA_4_ or 2′F-cA_4_ were measured by dynamic light scattering using the DynaPro NanoStar system (Wyatt Technology). The experiments were performed at 20°C. Samples containing CalpL at a final concentration of 86.6 μM (5 mg/ml) were prepared in 20 mM Tris–HCl pH 8.0, 50 mM NaCl, mixed with an equimolar amount of 2′F-cA_4_ or cA_4_ and centrifuged at 15 000 × *g* for 15 min to remove aggregates before each measurement. For each condition, three measurement cycles including each 20 single data acquisitions were performed. The acquisition time was set to 3 s. All experiments were performed in triplicates. To determine the significance of the radii difference between samples, a two-tailed unpaired t-test was performed (*P* values are included in the corresponding figures).

### Native PAGE

Native PAGE was performed to visualize oligomerization across the different CalpL mutants. For the assay, 15 μM of the CalpL variant was incubated with 5-fold molar excess of 2′F-cA_4_ or cA_4_ in the presence of 0.5 mM of the amine-reactive crosslinking agent bissulfosuccinimidyl suberate (BS3). The reactions were incubated for 30 min at 37°C, mixed with native PAGE sample loading buffer, loaded on a 10% Tris-glycine native PAGE gel and run at 40 mA/gel for 60 min. Gels were analyzed by Coomassie staining.

### Structural modelling

To construct a simple model of a cA_4_ induced oligomer of CalpL, two individual cA_4_ bound CalpL monomers (PDB-ID: 8b0r) were superimposed on the dimeric SAVED-CHAT structure (PDB-ID: 8tl0) with PyMOL (www.pymol.org). This led to a model with some clashes between the two protomers. The CHARMM-GUI (27) and GROMACS (28) were then used to perform an energy minimization of the stacked SAVED domains, including the sandwiched cA_4_ molecule. The resulting model was very similar to a model produced by AlphaFold3 (29) including 2 copies of CalpL and 12 AMP ligands (Supplementary Figure S5).

### SPR measurements

SPR measurements were performed on a Series S CM5 sensor chip using the Biacor™ 8K instrument (Cytiva). The chip was pre-equilibrated with running buffer (20 mM Tris pH 8, 50 mM NaCl, 0.05% (*v/v*) Tween20) at 25°C. CalpL was immobilized by amine-coupling using 50 mM NaOH for conditioning, and a 1:1 (*v/v*) mixture of 0.1 M N-hydroxysuccinimide (NHS) and 0.1 M 3-(*N*,*N*-dimethylamino)propyl-*N*-ethylcarbodimide (EDC) for surface activation. Subsequently, the flow system was washed with 1 M ethanolamine (pH 8). For immobilization, protein solutions were diluted in acetate buffer (pH 5) to a final concentration of 1 μM. To analyze the effect of protein immobilization density on the cA_4_ binding affinity, final protein concentrations of 1 μM, 500 nM, 250 nM, 125 nM, 62.5 nM and 31.25 nM were used for the immobilization process. Immobilization was performed in running buffer on the surface of the second flow cell for 160 s, or 100 s at a flow rate of 10 μl/min and afterwards, free binding sites on the surface were saturated with 1 M ethanolamine (pH 8) for 7 min at a flow rate of 10 μl/min. Single-cycle kinetics were used as analysis method mode with injections of increasing concentrations of the analyte over both flow cells, the association time was set to 120 s and a the dissociation time to 600 s at a flow rate of 30 μl/min. For A_2_ and A_4_, a concentration series of 0.26, 0.78, 2.33, 7, 21, 63, 189 and 567 nM was used. For cA_4_ and 2′F-cA_4_, a concentration series of 0.086, 0.26, 0.78, 2.33, 7, 21, 63 and 189 nM was used. Data were collected at a rate of 10 Hz, and double-referenced by blank cycles and reference flow cell subtraction. Binding parameters were obtained from the kinetic binding measurements using a 1:1 interaction model, using the Biacore^™^ Insight Evaluation Software (Cytiva).

## Results

### CalpL is a ring nuclease that degrades cA_4_ into two A_2_ units

To detect a possible ring nuclease activity of CalpL, we incubated CalpL with a 10-fold molar excess of cA_4_ and incubated the sample for 120 min at 60°C, corresponding to the growth temperature of *Sulfurihydrogenibium* spp. An HPLC analysis of the sample clearly showed that the substrate peak had disappeared and a new peak with approximately 2-fold stronger intensity eluting at earlier retention times was observed (Figure 1C). This product peak was identified as linear di-AMP (A_2_) by comparison with HPLC standards and mass spectrometry (Supplementary Figure S1, Supplementary Table S1).

In previous experiments, we identified cA_4_ as the activator of the Lon protease CalpL. SPR experiments revealed that CalpL binds cA_4_ with much higher affinity than other cOAs (16). Nevertheless, to a small extent, the protease activity of CalpL was also stimulated by other cOAs (cA_3_, cA_5_, cA_6_). While this activation by other cOAs is very likely not of any physiological relevance, it is interesting from a mechanistic point of view, because the activation mechanism appears to be ‘flexible’ enough to accommodate the different sizes of the cOAs. We thus wondered whether the CalpL ring nuclease activity is specific towards cA_4_ and performed nuclease assays using different cOAs (cA_3_, cA_5_, cA_6_) as substrates (Figure 1C, Supplementary Figure S1b–d). Interestingly, we observed significant cleavage of cA_3_ and cA_5_ within 120 min at 60°C, whereas cA_6_ was degraded only to a minor extent. For cA_3_, we observed an almost complete conversion into two cleavage products, which were identified as 2′,3′-cAMP (A_1_>P) and A_2_ by comparison with HPLC standards. For cA_5_, we observed an incomplete cleavage of cA_5_ to A_2_>P (5′-ApAp with a cyclic 2′,3′ phosphate), A_2_ and 2′,3′-cAMP. For cA_6_, we observed traces of A_2_>P, A_2_ and 2′,3′-cAMP, however most of the substrate remained uncleaved.

By analogy with *Sis*0455 (24), a member of the small standalone ring nuclease family, we wondered, whether the 2′-OH group of the cOA might be the nucleophile of the reaction. To test this, we performed the same experiment with the cA_4_ derivative 2′F-cA_4_ containing fluoro modifications in all ribose 2′ positions (Figure 1D). In this case, no substrate cleavage was observed within 120 min at 60°C, suggesting that the nuclease reaction indeed proceeds via positioning of the 2′-OH of the ribose for the nucleophilic attack.

**Figure 2.**
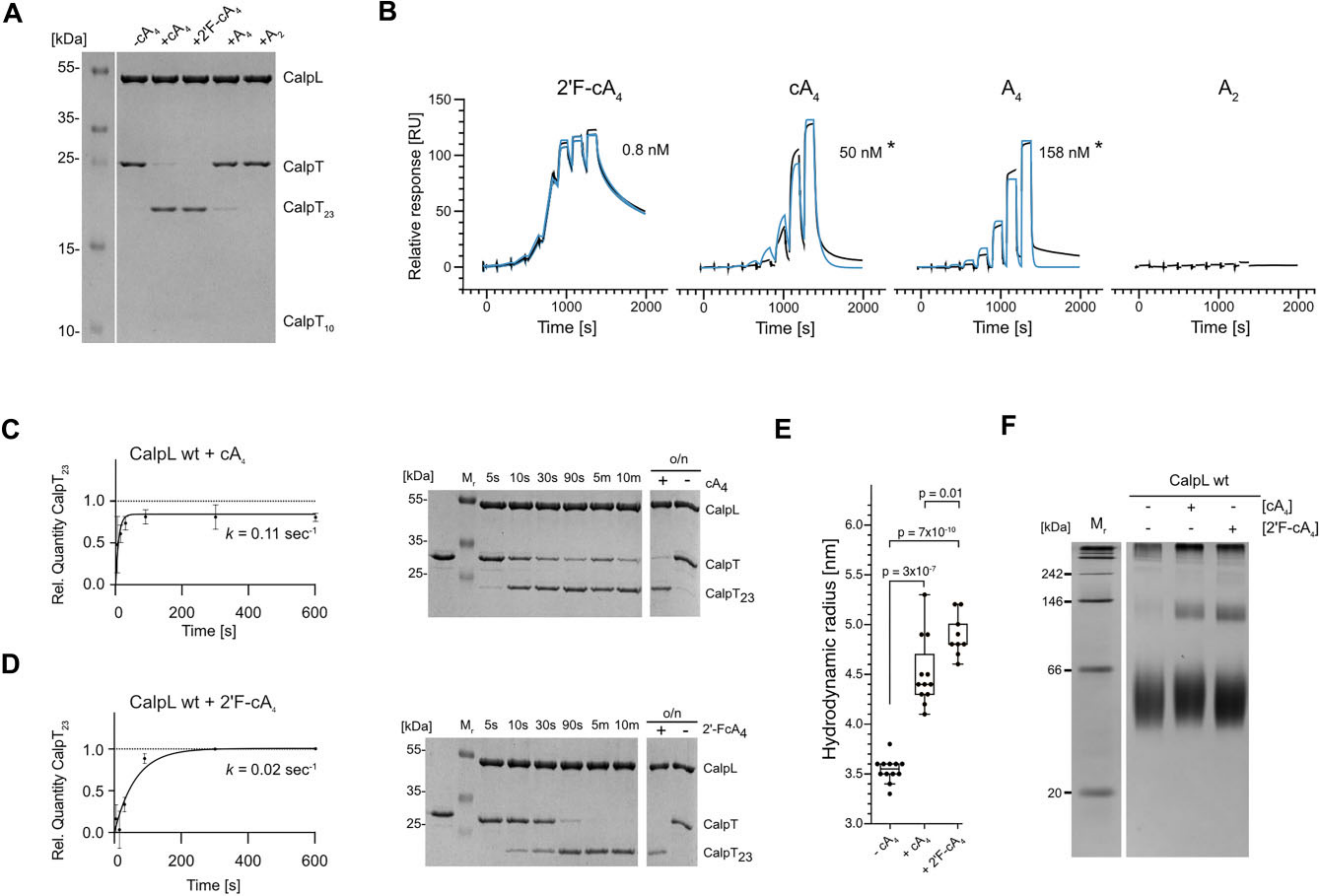
Degradation of cA_4_ limits the protease activity. (**A**) Coomassie-stained 15% SDS-PAGE analysis of CalpL protease activity assay showing the amount of CalpT cleavage upon addition of cA_4_, 2′F-cA_4_, A_2_ or A_4_. For the assay, 3 μM of CalpL and CalpT were mixed with 3 μM of the respective adenylate compound and incubated for 120 min at 60°C. (**B**) Single-cycle kinetics SPR measurements of 2′F-cA_4_, cA_4_, A_4_ and A_2_ binding to immobilized CalpL. The asterisk symbol (*) indicates the apparent *K*_D_ value determined for cA_4_ and A_4_ using the CM5 SPR setup. (**C**) Quantification of CalpT cleavage at 37°C for the indicated time periods of incubation of CalpL and CalpT with cA_4_ as described for (A). Values of all time points ranging from 5 s to 720 min were used for fit calculation. Right: Example of Coomassie-stained 15 % SDS-PAGE gel used for quantification. (**D**) same as in (B) but in the presence of 2′F-cA_4_. Experiments were performed in triplicates and error bars represent mean ± SD. (**E**) Hydrodynamic radii of CalpL determined at a sample concentration of 86 μM (5 mg/ml) in the presence or absence of 86 μM cA_4_ or 2′F-cA_4_. All DLS experiments were performed in triplicates. Bars display median and interquartile range; *p*-values for two-tailed t-tests are indicated. (**F**) Coomassie-stained 10% native PAGE analysis of CalpL oligomerization experiments. For the assay, 15 μM of CalpL was incubated with (+) or without (–) 75 μM cA_4_ or 2′F-cA_4_ for 30 min at 37°C in the presence of 0.5 mM bissulfosuccinimidyl suberate (BS3).

To assess the impact of CalpL nuclease activity on the CalpL/T/S cascade, we tested the cA_4_ degradation products for activation of the CalpL protease activity. As expected, we observed a strong proteolytic cleavage of CalpT in the presence of cA_4_ within 120 min at 60°C (Figure 2A). Whereas minor traces of uncleaved CalpT remained upon incubation with cA_4_, addition of the non-hydrolysable 2′F-cA_4_ resulted in a complete substrate cleavage. Substitution of cA_4_ with A_4_ (with a 3′ phosphate) led to a strongly decreased but detectable amount of CalpT cleavage as judged by Coomassie-stained SDS-PAGE. For A_2_ (with a 3′ phosphate), we did not observe CalpT cleavage, demonstrating that cA_4_ degradation by CalpL limits the protease activity. This observation aligns with a decrease in affinity of CalpL towards A_4_ and A_2_ compared to cA_4_, as observed in SPR spectroscopy experiments (Figure 2B).

We were puzzled that the *K*_D_ value for cA_4_ (50 nM) was much lower than both, the *K*_D_ values determined for 2′F-cA_4_ (1 nM) and the *K*_D_ for cA_4_ of ∼1 nM that had been determined in our previous study (16). We analyzed this in detail and found that the difference was caused by the different CalpL immobilization strategies used in the two studies. In our previous study, we used streptavidin coated SPR chips to immobilize CalpL via a biotin-labelled cysteine at the bottom side of the SAVED domain, i.e. opposite of the cA_4_ binding site. In the present study, due to a very large set of single mutants, we used stochastic immobilization via amine-coupling to CM5 chips. We suspected that the biotin anchor in our previous study had blocked any oligomerization of CalpL on the chip and that this might also have inhibited the nuclease activity. To test this, we performed an experiment, in which we immobilized a dilution series of CalpL on different flow cells of a CM5 chip and measured the affinity for cA_4_ and 2′F-cA_4_. Since the chances of oligomer formation would be lower when less CalpL was present on the chip, we hypothesized that the apparent affinity for cA_4_ would increase. In contrast, the affinity for the non-hydrolysable 2′F-cA_4_ would remain constant. Indeed, the SPR titration experiment confirmed a strong correlation between the quantity of immobilized CalpL and the resultant K_D_ values for cA_4_ binding (Supplementary Figure S2a–f). With decreasing amounts of immobilized CalpL, the apparent affinity towards cA_4_ asymptotically reached the value of ∼1 nM, matching the value determined previously using the biotin-coupling approach. As expected, the affinity towards 2′F-cA_4_ remained unaffected by the immobilization rate. From these observations, we concluded that the affinities for cA_4_ and most likely also for A_4_ that were measured with a CM5 SPR chip are ‘apparent’ *K*_D_ values and that the measurements with 2′F-cA_4_ can be seen as a surrogate for the first contact of cA_4_ with CalpL, i.e. before the nuclease reaction occurs. Also, these experiments strongly indicated that the oligomerization of CalpL plays an important role in the nuclease activity.

Based on the observation that the protease activity of CalpL is strongly reduced upon linearization of cA_4_, we speculated, whether 2′F-cA_4_ might further enhance the proteolytic activity of CalpL. To test this, we performed protease reaction time courses by incubating equimolar concentrations of CalpL and CalpT in the presence of either cA_4_ or 2′F-cA_4_ (Figure 2C, D). Further, we studied the oligomerization of CalpL in the presence of cA_4_ or 2′F-cA_4_ by determining the hydrodynamic radii using dynamic light scattering (DLS) (Figure 2E) and by bissulfosuccinimidyl suberate (BS3) crosslinking combined with native PAGE experiments (Figure 2F). Indeed, DLS analysis revealed a slightly increased hydrodynamic radius for CalpL of 4.9 ± 0.21 nm upon binding of 2′F-cA_4_, compared to 4.5 ± 0.35 nm upon addition of cA_4_. We interpret the differences in hydrodynamic radius as a change in the propensity of CalpL to form oligomers, depending on the cofactor. In the native PAGE experiments, this increase in hydrodynamic radius was matched by a strong increase in intensity of a band running at approx. 120–130 kDa, corresponding to a CalpL dimer. Further, despite a slower initial velocity (v_0_) of the protease reaction in the presence of 2′F-cA_4_, we observed complete consumption of CalpT after 5 min of incubation with 2′F-cA_4_ at 37°C, whereas activation with cA_4_ failed to induce full cleavage of CalpT even after overnight incubation (Figure 2C, D).

### Nuclease reaction time courses and identification of intermediates

**Figure 3.**
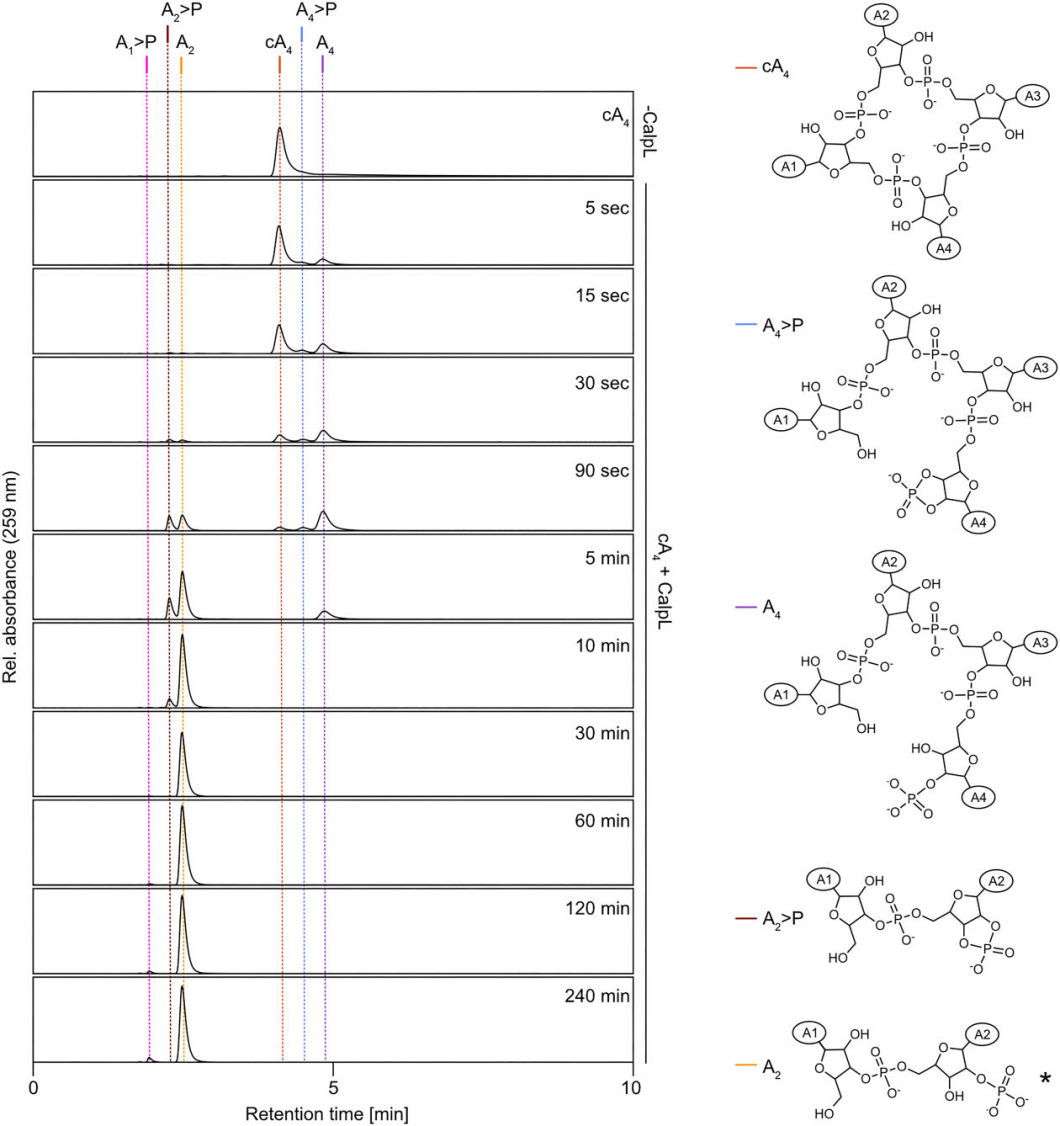
Time course of CalpL-mediated cA_4_ cleavage. Left: HPLC traces of nuclease reactions incubating 1.5 μM CalpL with 15 μM cA_4_ at 37°C for the indicated time periods. The vertical lines mark reaction intermediates and products that were identified by comparison with standard runs and/or mass spectrometry. Right: Structural formulas of the observed reaction intermediates. The adenosine base is represented by A_1_–A_4_ in ellipsoids. All HPLC traces are representative of at least three replicates. The asterisk symbol (*) indicates that hydrolysis of the cyclic 2′,3′-phosphate could yield products having the phosphate group at either the 2′ or 3′ position.

To follow the reaction resulting in the conversion of cA_4_ to A_2_, we performed time course experiments and analyzed the reaction products by HPLC/MS (Figure 3, Supplementary Figure S3, Supplementary Table S1). Due to the high speed of the ring nuclease reaction, we lowered the reaction temperature from 60°C to 37°C, resulting in a reduced activity of CalpL. The reactions were stopped by flash freezing in liquid nitrogen and thawed immediately before injection onto the column. Five seconds after the start of the reaction, two additional peaks with higher retention times appeared. Using MS, these were identified as A_4_ (larger peak, 4.4 min) and A_4_>P (smaller peak, 4.1 min). Within 30 seconds of incubation, a large fraction of cA_4_ was consumed and converted into A_4_, with only a minor A_4_>P fraction being present at all time points tested. In addition, two smaller peaks at much earlier retention times had appeared. MS analysis revealed that the peaks had the mass of linear A_2_ (2.4 min) and of A_2_>P (2.2 min). Over the next minutes, the A_2_>P intermediate was almost completely converted to A_2_. Note that we observed very small amounts of A_1_>P (1.9 min), but no A_3_>P, being formed after extended incubation periods. All assignments were confirmed by comparison with HPLC standards (Supplementary Figure S3). In summary, the CalpL ring nuclease reaction proceeds from cA_4_ via A_4_>P, A_4_, A_2_>P to A_2_.

### Residues at both the top- and bottom sides of the SAVED domain are involved in ring nuclease activity

**Figure 4.**
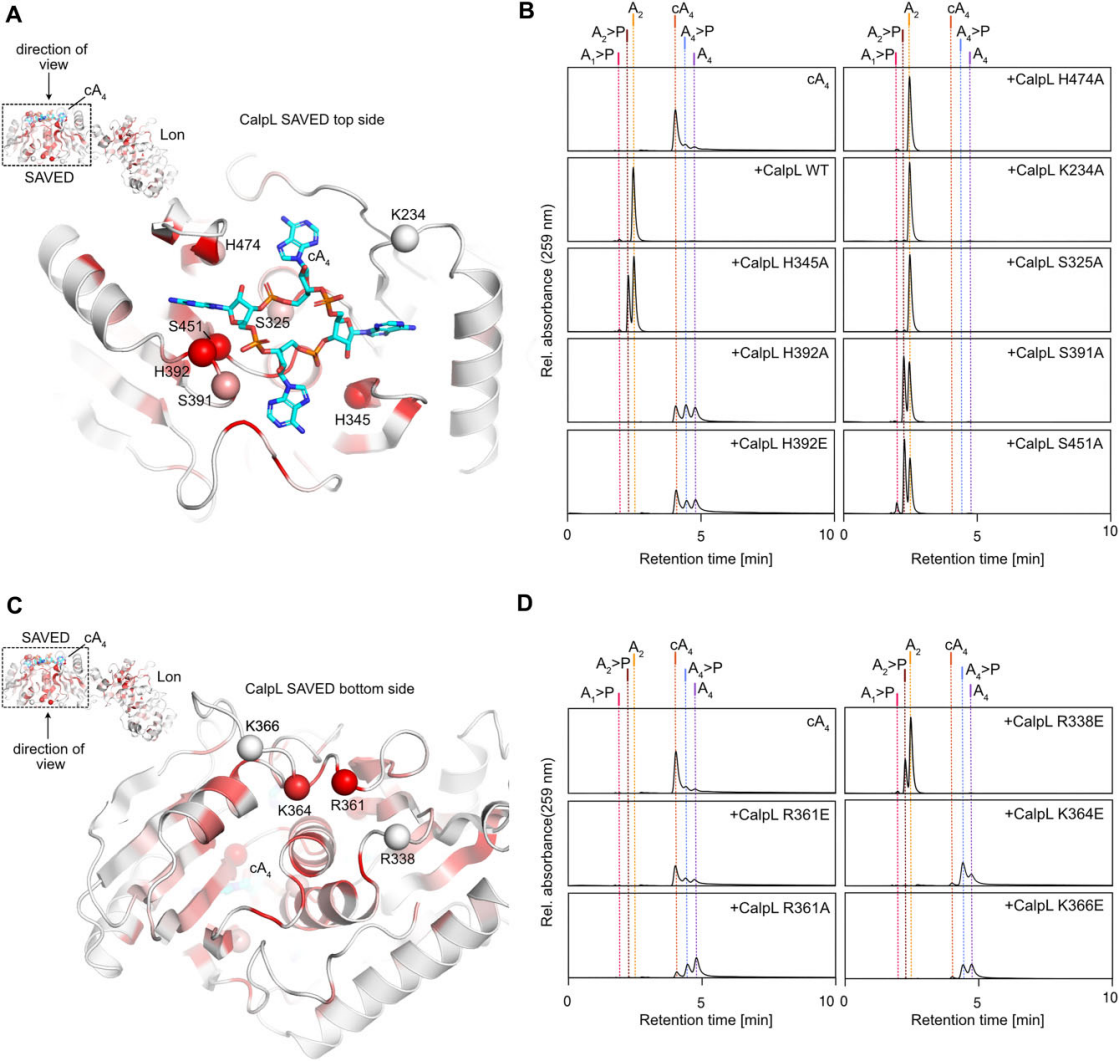
CalpL mutants in the cA_4_ binding site and on the bottom of the SAVED domain show impaired ring nuclease activity. (**A**) A cartoon model of the SAVED domain of CalpL (viewing direction with respect to the complete structure is indicated). The conservation of residues is mapped onto the structure (red: high conservation score, white low conservation score; The underlying alignment is provided as Supplementary Material). The cA_4_ molecule is shown in cyan sticks. Residues that were mutated are marked by a sphere at their Cα position. (**B**) HPLC analysis of ring nuclease reactions incubating 1.5 μM of the respective SAVED topside variant indicated in a) with 15 μM cA_4_ at 60°C for 120 min. (**C**) As in (A) but the bottom side of the SAVED domain is shown. (**D**) HPLC analysis of ring nuclease reactions performed as in (B) but of SAVED bottom side indicated in c). All HPLC traces are representatives of at least three replicates.

Considering the fact that cA_4_ is split into two A_2_ molecules, we hypothesized that the cleavage occurs at two opposing sites within the active site. Based on structural data obtained from the CalpL-cA_4_ complex (Figure 1A) combined with sequence alignments of CalpL homologs, we identified conserved side chains, which are located in the cA_4_ binding site and might be involved in the nuclease reaction: H345, H392, H474, S325, S391, S451 (Figure 4A).

To assess their contribution to the ring nuclease reaction, we mutated the conserved residues to alanine, either individually or in combination, and examined the cA_4_ cleavage reaction at 60°C by HPLC analysis (Figure 4B).

The strongest effect of nuclease attenuation was observed for mutants H392A and H392E. Here, we observed a slow conversion of cA_4_ to A_4_>P and A_4_, but we did not observe any production of di-adenylates after incubation for 120 min. Whereas mutation of either S325 or H474 to alanine did not affect the nuclease activity, H345A and S391A variants of CalpL showed a reduced ability to convert A_2_>P to A_2_ within the observed time frame. We suspected a potential cooperation of H345 and H392, however surprisingly, a combined mutation to alanine resulted in an enhanced nuclease activity compared to mutation of H392 alone (Supplementary Figure S4). Here, we observed production of A_2_>P and A_2_, with only a minor fraction of A_4_ present after incubation at 60°C for 120 min. Interestingly, we further observed production of a novel reaction product which was identified as A_3_>P by comparison with HPLC standards (Supplementary Figure S4).

Although not conserved, we considered K234, located directly opposed to H392, as a possible candidate for being involved in a coordinated cA_4_ cleavage. As judged by the reaction endpoint analysis, mutation of K234 to alanine did not influence the nuclease activity of CalpL. Similar to the H345A/H392A double mutant, a combined mutation of K234 and H392 to alanine enhanced the nuclease activity compared to the H392 single alanine mutant, and further resulted in the production of minor traces of A_3_>P. A combined alanine mutation of H345 and K234 did not enhance the nuclease attenuating effect observed for H345A alone and did not show production of tri-adenylate species.

In summary, of all residues inside the cA_4_ binding site, only residues in the vicinity of H392 showed a strong impact on the ring nuclease activity, suggesting that at least one cleavage reaction occurs at this position.

Puzzled by the persistence of nuclease activity despite systematic mutation of residues within the cA_4_ binding pocket, our attention was drawn to the high conservation of a positively charged patch on the bottom side of the SAVED domain (Figure 4C). Recent reports on CARF domain-containing ring nucleases and oligomerizing SAVED domains (17,30–33) prompted us to investigate whether conserved bottom side residues might be involved in the ring nuclease reaction when CalpL forms oligomers. We mutated the corresponding residues, either to alanine or glutamate, and assessed the reaction products via HPLC (Figure 4D). Strikingly, all bottom side mutants showed a significantly impaired nuclease activity, with none achieving a complete conversion of cA_4_ to A_2_ within 120 min at 60°C. While K364E and K366E variants showed a minimal production of A_4_>P and A_4_ within the observed time frame, mutation of R361 to glutamate resulted in a complete disruption of the nuclease activity. Interestingly, an R361A variant retained basal levels of nuclease activity and showed production of A_4_>P and A_4_.

As suspected from the SPR results described above, these observations indicated a strong link between oligomerization and nuclease activity; however, the exact allocation of active site residues is not trivial.

### Interdependence of the nuclease and protease activities of CalpL

**Figure 5.**
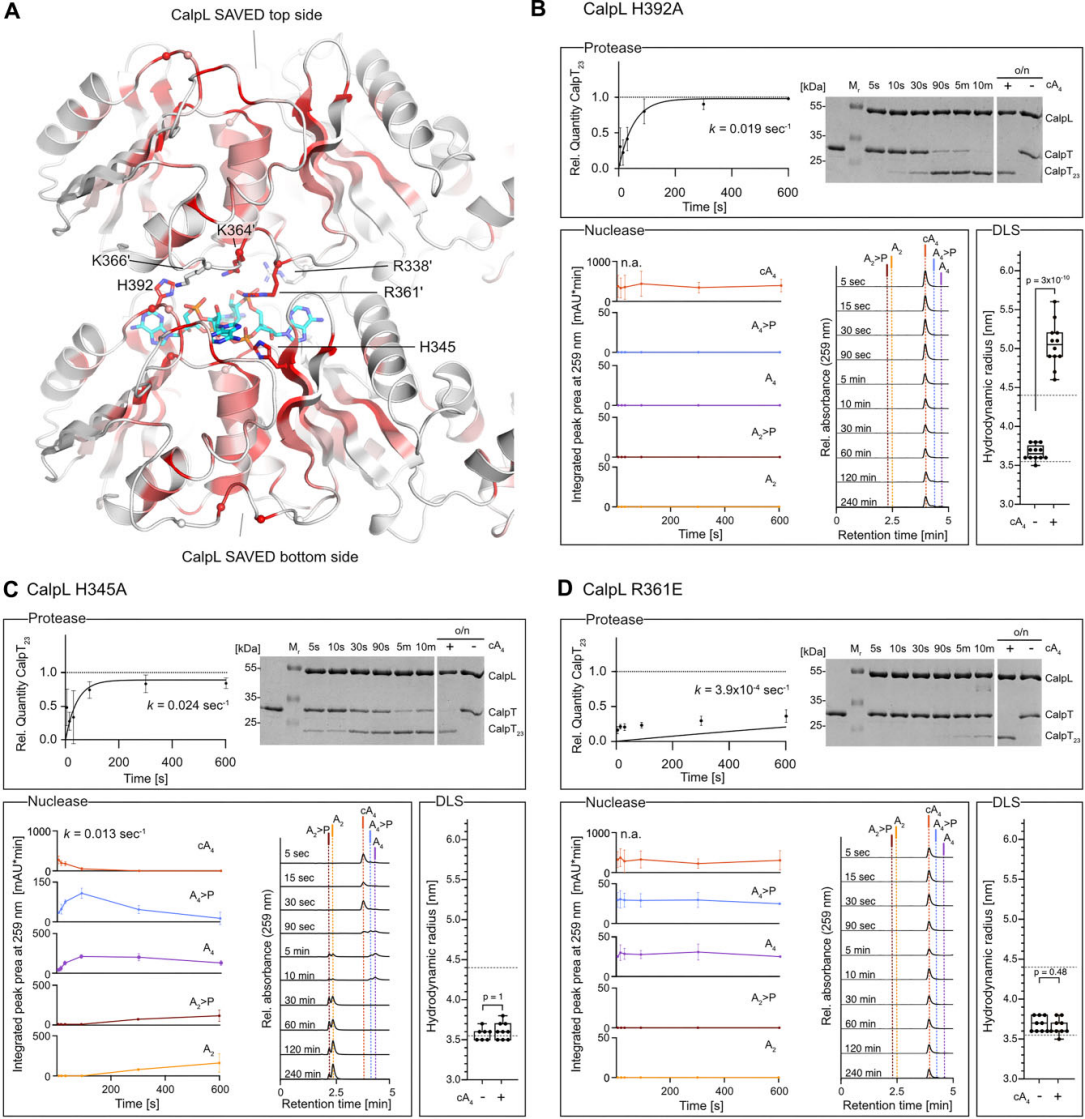
Interdependence of ring nuclease and protease activities of CalpL. (**A**) An energy-minimized model for cA_4_-mediated oligomerization of CalpL based on the SAVED-CHAT structure (PDB-ID: 8tl0, (17)) generated with GROMACS (28). The cA_4_ molecule is shown as cyan sticks. (**B**–**D**) Upper panels: Quantification of CalpT cleavage by incubating 3 μM of the respective CalpL mutant with 3 μM CalpT at 37°C for the indicated time points. Values of all time points ranging from 5 s to 720 min were used for fit calculation. For mutant R361E, the fit was not of high quality for the early reaction timepoints but of good quality for the complete experiment. Therefore, a graph covering the complete experiment is shown in Supplementary Figure S7a). A Coomassie-stained 15% SDS-PAGE gel used for quantification is included for reference. Left lower panels: Quantification of nuclease reaction intermediates and products generated upon incubation of 1.5 μM CalpL and 15 μM cA_4_ at 37°C for the indicated time points. HPLC traces used for quantification by peak area integration at 259 nm are shown exemplary. Right lower panels: Hydrodynamic radii of CalpL determined at a sample concentration of 86 μM (5 mg/ml) in the presence or absence of 86 μM cA_4_. Dashed lines mark the hydrodynamic radii of wildtype CalpL with (4.5 ± 0.35 nm) and without (3.5 ± 0.12 nm) cA_4_. All experiments were performed in triplicates. Bars of DLS data display median and interquartile range; *P*-values for two-tailed *t*-tests are indicated.

To understand how oligomerization could trigger the enzymatic activities of CalpL, we generated a simple model of a CalpL oligomer based on the stacked SAVED domains of the recently published SAVED-CHAT structure (Figure 5A) (17). Although the model cannot be used for a detailed structural analysis, it nicely illustrates that conserved residues of both, the CalpL bottom- and top sides, including R338, R361, K364 and K366 can potentially contact the sandwiched cA_4_ molecule, rationalizing their influence on the ring nuclease activity. A very similar model of a CalpL oligomer was produced with the recently released AlphaFold3 server in the presence of AMP ligands that were used as a surrogate for cA_4_ (Supplementary Figure S5). Note that our model also resembles a very recent cryo-EM structure of the CalpL homolog CCaCalpL (20).

Activation of the ring nuclease upon oligomer formation and protease activation constitutes an elegant mechanism to limit the protease activity. Consequently, we wondered if and how a reduced nuclease activity, such as observed for top side residue H392A or bottom side residues R361A/E, K364E and K366E might influence the protease activity of CalpL. To study this, we performed protease time course experiments incubating the different CalpL variants and the protease substrate CalpT and quantified the protease activities using Coomassie-stained SDS-PAGE (Figure 5B–D, Supplementary Figure S6, Supplementary Figure S7, Supplementary Table S2). For each mutant, we assessed its propensity to form CalpL oligomers by dynamic light scattering (DLS) (Figure 5B–D, Supplementary Figure S8) and by performing native PAGE experiments (Supplementary Figure S9). Further, we confirmed the cA_4_-, A_4_- and A_2_- binding capacities of all mutants by performing SPR experiments (Supplementary Figure S10, Supplementary Table S3).

DLS analysis revealed a strongly enhanced propensity for cA_4_-induced oligomerization for the nuclease-deficient H392A topside mutant as judged by a hydrodynamic radius of 5.1 ± 0.28 nm, similar to the hydrodynamic radius of 4.9 ± 0.21 nm observed upon 2′F-cA_4_-induced oligomerization of the wildtype protein shown in Figure 2. Analogous to the activation of wildtype CalpL with 2′F-cA_4_, the protease reaction of the H392A variant showed a reduced initial velocity v_0_ but achieved full cleavage of CalpT within 10 min (Figure 5B, Supplementary Table S2). Interestingly, the SPR experiments revealed that H392A has a very high affinity for A_4_, explaining that the mutant behaves similar as the wildtype in the presence of the nuclease-inert 2′F-cA_4_ (Supplementary Figure S10, Supplementary Table S3).

The CalpL H345A variant, which had an impaired capacity to convert A_2_>P to A_2_ in the reaction endpoint experiment, showed wild-type like cA_4_ cleavage in the time-course experiment (Figure 5C, Supplementary Table S2). However, the H345A mutant had a decreased protease activity (Supplementary Table S2) with a minor fraction of CalpT remaining uncleaved after overnight incubation. Further, we did not observe cA_4_-induced oligomerization of mutant H345A by DLS, as evident from a similar hydrodynamic radius in the presence (3.6 ± 0.11 nm) and absence (3.6 ± 0.13 nm) of cA_4_. The SPR data showed that nuclease-inert 2′F-cA_4_ (and thus cA_4_) binds much weaker to this mutant and we observed a very fast dissociation compared to wildtype CalpL, presumably destabilizing any oligomers that are formed. To test whether sporadic oligomerization events took place that could explain the remaining protease activity, we performed BS3 crosslinking experiments in combination with native PAGE. The rational was that the crosslinking reagent would immediately trap any oligomers that were formed. As shown in Supplementary Figure S9, this was indeed the case, as the addition of cA_4_ and 2′F-cA_4_ resulted in a strong increase in dimeric species compared to a control reaction containing no cA_4_.

Intriguingly, for the H474A mutant, we observed a strong ring nuclease activity (Supplementary Figure S6d, Supplementary Table S2) (Supplementary Figure S6), which, in contrast to that of the wildtype protein, did not correlate with an early termination of the protease reaction. Here, we observed a complete substrate cleavage within 10 min despite an almost complete consumption of A_4_ after 5 min. The oligomerization behavior, however, remained unaltered (Supplementary Figure S6). Also here, the SPR data provided a potential explanation for the observed effects. We found that the affinity of mutant H474A for 2′F-cA_4_ (and therefore, as explained above, presumably for uncleaved cA_4_) was approx. 4-times higher than the affinity of wildtype CalpL for 2′F-cA_4_ (Supplementary Figure S10, Supplementary Table S3).

In the case of the nuclease-dead mutant R361E, we observed a strong decrease in protease activity (Figure 5d), correlating with the absence of cA_4_-induced oligomerization as judged by DLS. Conversely, mutant R361A, which showed basal levels of nuclease activity, exhibited an only slightly reduced proteolytic activity, while showing marginal cA_4_-induced oligomerization (Supplementary Figure S6f). We analyzed both mutants via crosslinked native PAGE, which confirmed the weak propensity of these mutants to oligomerize, explaining the remaining protease activity (Supplementary Figure S9). We speculate that the geometry of the oligomers is influenced by the type of amino acid that is introduced at position R361, explaining the differences in protease activity.

For mutants K364E and K366E, which showed only marginal cA_4_ to A_4_ conversion upon incubation at 60°C for 120 min, we observed no cA_4_ cleavage upon incubation at 37°C for 240 min. In the protease time course experiments, both mutants showed a significant reduction in proteolytic activity, which was more pronounced for K366E than for K364E (Supplementary Table S2, Supplementary Figure S6g, h). Further, the K364E variant showed wildtype-like oligomerization as judged by a hydrodynamic radius of 4.64 ± 0.142 nm in the presence of cA_4_, compared to mutant K366E which did not show a response to cA_4_ as judged by a hydrodynamic radius of 3.66 ± 0.073 nm in the presence of cA_4_ (Supplementary Figure S8). Again, these observations were also reflected in our crosslinked native PAGE experiments, where only very faint oligomerization was observed for CalpL K366E (Supplementary Figure S9).

### Cooperation of SAVED domains during ring nuclease and protease reactions

**Figure 6.**
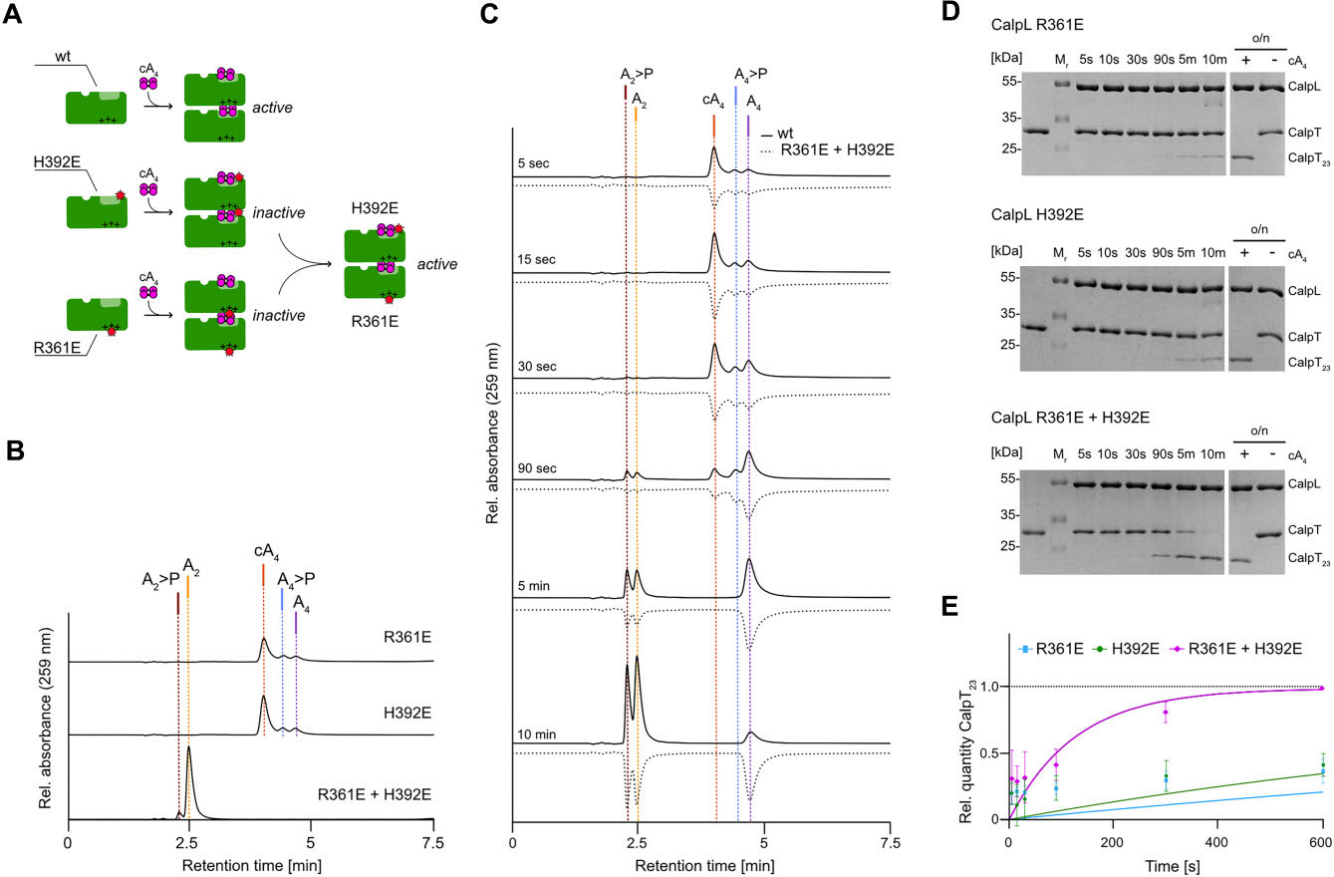
SAVED domains cooperate during ring nuclease and protease reactions. (**A**) Schematic representation of the CalpL activity reconstitution assay. (**B**) HPLC analysis of ring nuclease endpoint reactions incubating 1.5 μM of the respective CalpL mutant or an equimolar mixture of both mutants (heterodimer) with 15 μM cA_4_ for 120 min at 60°C. (**C**) Same as in (b) but performed as a time course experiment incubating the reactions at 37°C for the indicates time points. The traces for the heterodimer are mirrored across the time axis for illustration purposes. (**D**) Representative 15% Coomassie-stained SDS-PAGE gels of protease time course assays incubating 3 μM of the indicated mutant or 3 μM of the R361E/H392E heterodimer with equimolar amounts of CalpT and cA_4_ at 37°C for the indicated time points. (**E**) Quantification of protease time courses shown exemplary in (D). Values of all time points ranging from 5 s to 720 min were used for fit calculation. For the individual mutants, the fit was not of high quality for the early reaction timepoint but of good quality for the complete experiment. Therefore, a graph covering the complete experiment is shown in Supplementary Figure S7a, b). All experiments were performed in triplicates.

Having identified key residues on both the SAVED top side and bottom side which lead to the disruption of ring nuclease and protease activities upon mutation, we sought to investigate a potential synergistic action of these residues during cA_4_ cleavage. Similar to Smalakyte *et al.* (20), we combined the nuclease-dead SAVED topside mutant H392E and the SAVED bottom side mutant R361E to test for reconstitution of an active heterodimer from two inactive CalpL monomers (Figure 6A). To evaluate the ring nuclease activity of the H392E/R361E heterodimer, we performed ring nuclease endpoint experiments and analyzed the reactions using RP-HPLC (Figure 6B). Whereas the individual mutants did not show any nuclease activity upon incubation with cA_4_ for 120 min at 60°C, we observed a complete conversion of cA_4_ to the final reaction product A_2_ upon co-incubation with an equimolar mixture of CalpL R361E and H392E. We further performed time-course assays to compare the nuclease activity of the R361E/H392E heterodimer to the wildtype variant and observed that the heterodimer showed an only slightly decreased di-adenylate production compared to the wildtype protein (Figure 6C).

Interestingly, the rescue of enzymatic function upon reconstitution of the active heterodimer was not only observed for the ring nuclease activity, but was similarly detected for the proteolytic activity of CalpL (Figure 6D). Whereas the individual mutants R361E and H392E induced only minor cleavage of CalpT within 10 min, an equimolar mixture of both mutants achieved almost full substrate cleavage within the same time frame. As mutant H392E exhibited a wildtype-like oligomerization behavior in response to cA_4_, we speculate that charge-reversion of residues within the ring nuclease active site and thus oligomerization interface affects the precision of oligomer assembly, a phenomenon which does not necessarily correlate with the overall propensity to oligomerize. Reconstituting the minimal wildtype-like active site alleviated the loss of protease activity, indicating that a native CalpL active site is crucial for both protease and ring nuclease activities of CalpL.

## Discussion

Here, we have characterized the ring nuclease reaction of the CalpL SAVED domain. The protein rapidly degrades its activator, the cyclic oligonucleotide cA_4_ to linear A_2_ via several intermediates with cyclic phosphate groups. We did not observe any indications of a metal dependency of the reaction and the fact that the chemical analog 2′F-cA_4_ was not degraded, together with the observation of cyclic phosphate intermediates are strong indicators of the 2′-OH group of the cA_4_ molecule being the nucleophile of the reaction.

Our previously determined structure of CalpL in complex with cA_4_ allows us to speculate about the location of the scissile bonds in the cA_4_ molecule with respect to the surrounding protein surface. We found a strong effect on the rate of the A_2_>P to A_2_ reaction when residues near H392 were mutated. This indicates that at least one cleavage reaction takes place at this site. Since the final product is linear A_2_, a second cleavage must occur and the logical position for this reaction would be exactly opposite of H392. However, we did not observe strong effects on the nuclease reaction when residues at this position in the cA_4_ binding site were mutated, such as K234A. The reconstitution of an active CalpL heterodimer from the two nuclease-dead variants H392E and R361E indicates that residues from the bottom side of CalpL oligomers are involved in the formation of a composite ring nuclease active site. However, our data do not allow us to distinguish, whether they play a catalytic or merely a structural role.

Due to the extensive accumulation of A_4_ during the nuclease reaction time courses (an excess of cA_4_ was used in the experiments), we can conclude that A_4_ is released from the cA_4_ binding site and reenters the active site for the second cleavage. While this seems ineffective, the surprisingly high affinity of CalpL for A_4_ (Supplementary Figure S10) explains why the reaction still progresses very fast. The fact that only very small amounts of A_1_>P were observed indicates that the A_4_ molecule is aligned in the active site such that the second cleavage takes place opposite of the first one.

We found that the ring nuclease activity of CalpL is severely decreased for mutants that also show a decreased capability to form stable CalpL oligomers, such as R361A/E, and K366E. This would ensure that the ring nuclease activity is only fully activated when the cA_4_ concentration is high enough to allow the formation of oligomers and would thereby avoid a decrease in sensitivity by premature hydrolysis of cA_4_ molecules. As our data suggests that these mutants undergo sporadic oligomerization, we conclude that the remaining nuclease activity of these mutants is due to such rare events. A high-resolution structure of a stacked SAVED domains of CalpL would certainly be helpful to further unravel the mechanism of the ring nuclease reaction. In previous SAXS experiments (16), we observed indications of a staggered arrangement of CalpL monomers in solution, though at low resolution. This is quite different to the non-staggered arrangement found in the SAVED-CHAT- and CCaCalpL structures and from the non-staggered AF3 model (17,20) (Supplementary Figure S5). Our model based on the latter structures can rationalize the effects of bottom side mutants analyzed in this study very well. However, since the cA_4_ binding site is pseudo C2 symmetric and the SAXS data were of high quality, it might be that both arrangements can occur in solution, especially considering the fact that CalpL is normally tightly bound to the CalpT and CalpS proteins.

Studies exploring the molecular mechanisms of type III CRISPR associated ring nucleases have described a diverse array of nuclease activation processes. These mechanisms encompass intricate conformational changes within the cOA binding site, leading to an alignment of the active site (25,34), as well as global structural rearrangements including multimerization, where individual nucleases assemble to form a composite active site from previously isolated regions of the protein (35). CalpL is the first protein with a SAVED domain to show a ring nuclease activity and appears to be a mix of features observed in other ring nucleases.

Most likely, the CalpL nuclease activity will lead to a self-quenching effect of the antiviral response, as observed for other type III CRISPR systems. This indicates that the CalpL/T/S pathway is not aimed at an abortive infection but rather at a reversible adjustment of the organism to a phage attack where even a small amount of released CalpS might be enough to appropriately fine tune the transcriptional levels of the cell. Alternatively, the ring nuclease activity might also play a role in setting up an activation threshold such that basal levels of cA_4_ are quickly degraded. However, since the nuclease activity appears to go hand-in-hand with oligomer formation and thereby the activation of the protease activity of CalpL the self-quenching effect appears to be the more likely explanation for its ring nuclease activity.

The CalpL protein combines a staggering number of functionalities in a relatively small protein. The inducible protease activity makes it a potent tool for biotechnological applications, such as for instance for CRISPR-based sensing assays, where the protease activates a response mechanism. Due to its negative impact on the protease activity, the ring nuclease activity is a disadvantage for such applications. Therefore, our discovery that it can be deactivated while maintaining the protease activity is of high interest for such efforts.

## Supplementary Material

gkae676_Supplemental_Files

## Data Availability

The model in Figure 5 is available as a Supplementary File.

## References

[1] Tal N. , Sorek R. SnapShot: bacterial immunity. Cell. 2022; 185:578–578. 35120666 10.1016/j.cell.2021.12.029

[2] Agapov A. , Baker K.S., Bedekar P., Bhatia R.P., Blower T.R., Brockhurst M.A., Brown C., Chong C.E., Fothergill J.L., Graham S. et al. Multi-layered genome defences in bacteria. Curr. Opin. Microbiol. 2024; 78:102436. 38368839 10.1016/j.mib.2024.102436

[3] Gasiunas G. , Sinkunas T., Siksnys V. Molecular mechanisms of CRISPR-mediated microbial immunity. Cell. Mol. Life Sci. 2014; 71:449–465. 23959171 10.1007/s00018-013-1438-6 PMC3890593

[4] Sasnauskas G. , Siksnys V. CRISPR adaptation from a structural perspective. Curr. Opin. Struct. Biol. 2020; 65:17–25. 32570107 10.1016/j.sbi.2020.05.015

[5] Wang J.Y. , Pausch P., Doudna J.A. Structural biology of CRISPR–Cas immunity and genome editing enzymes. Nat. Rev. Microbiol. 2022; 20:641–656. 35562427 10.1038/s41579-022-00739-4

[6] Jinek M. , Chylinski K., Fonfara I., Hauer M., Doudna J.A., Charpentier E. A programmable dual-RNA-guided DNA endonuclease in adaptive bacterial immunity. Science. 2012; 337:816–821. 22745249 10.1126/science.1225829 PMC6286148

[7] Gasiunas G. , Barrangou R., Horvath P., Siksnys V. Cas9–crRNA ribonucleoprotein complex mediates specific DNA cleavage for adaptive immunity in bacteria. Proc. Natl. Acad. Sci. U.S.A. 2012; 109:E2579–E2586. 22949671 10.1073/pnas.1208507109 PMC3465414

[8] Samai P. , Pyenson N., Jiang W., Goldberg G.W., Hatoum-Aslan A., Marraffini L.A. Co-transcriptional DNA and RNA cleavage during type III CRISPR-Cas immunity. Cell. 2015; 161:1164–1174. 25959775 10.1016/j.cell.2015.04.027 PMC4594840

[9] Hale C.R. , Cocozaki A., Li H., Terns R.M., Terns M.P. Target RNA capture and cleavage by the cmr type III-B CRISPR–Cas effector complex. Genes Dev. 2014; 28:2432–2443. 25367038 10.1101/gad.250712.114 PMC4215187

[10] Staals R.H.J. , Zhu Y., Taylor D.W., Kornfeld J.E., Sharma K., Barendregt A., Koehorst J.J., Vlot M., Neupane N., Varossieau K. et al. RNA targeting by the type III-A CRISPR-Cas csm complex of thermus thermophilus. Mol. Cell. 2014; 56:518–530. 25457165 10.1016/j.molcel.2014.10.005 PMC4342149

[11] Kazlauskiene M. , Kostiuk G., Venclovas Č., Tamulaitis G., Siksnys V. A cyclic oligonucleotide signaling pathway in type III CRISPR-Cas systems. Science. 2017; 357:605–609. 28663439 10.1126/science.aao0100

[12] Niewoehner O. , Garcia-Doval C., Rostøl J.T., Berk C., Schwede F., Bigler L., Hall J., Marraffini L.A., Jinek M. Type III CRISPR-Cas systems produce cyclic oligoadenylate second messengers. Nature. 2017; 548:543–548. 28722012 10.1038/nature23467

[13] Athukoralage J.S. , White M.F. Cyclic oligoadenylate signalling and regulation by ring nucleases during type III CRISPR defence. RNA. 2021; 27:855–867. 33986148 10.1261/rna.078739.121 PMC8284326

[14] Jia N. , Jones R., Sukenick G., Patel D.J. Second messenger cA4 formation within the composite Csm1 Palm pocket of type III-A CRISPR-Cas Csm complex and its release path. Mol. Cell. 2019; 75:933–943. 31326272 10.1016/j.molcel.2019.06.013 PMC6731140

[15] Makarova K.S. , Anantharaman V., Grishin N.V., Koonin E.V., Aravind L. CARF and WYL domains: ligand-binding regulators of prokaryotic defense systems. Front. Genet. 2014; 5:102. 24817877 10.3389/fgene.2014.00102 PMC4012209

[16] Rouillon C. , Schneberger N., Chi H., Blumenstock K., Vela S.D., Ackermann K., Moecking J., Peter M.F., Boenigk W., Seifert R. et al. Antiviral signalling by a cyclic nucleotide activated CRISPR protease. Nature. 2023; 614:168–174. 36423657 10.1038/s41586-022-05571-7

[17] Steens J.A. , Bravo J.P.K., Salazar C.R.P., Yildiz C., Amieiro A.M., Köstlbacher S., Prinsen S.H.P., Andres A.S., Patinios C., Bardis A. et al. Type III-B CRISPR-Cas cascade of proteolytic cleavages. Science. 2024; 383:512–519. 38301007 10.1126/science.adk0378 PMC11220425

[18] Hu C. , Beljouw S.P.B.v., Nam K.H., Schuler G., Ding F., Cui Y., Rodríguez-Molina A., Haagsma A.C., Valk M., Pabst M. et al. Craspase is a CRISPR RNA-guided, RNA-activated protease. Science. 2022; 377:1278–1285. 36007061 10.1126/science.add5064 PMC10041820

[19] Strecker J. , Demircioglu F.E., Li D., Faure G., Wilkinson M.E., Gootenberg J.S., Abudayyeh O.O., Nishimasu H., Macrae R.K., Zhang F. RNA-activated protein cleavage with a CRISPR-associated endopeptidase. Science. 2022; 378:874–881. 36423276 10.1126/science.add7450 PMC10028731

[20] Smalakyte D. , Ruksenaite A., Sasnauskas G., Tamulaitiene G., Tamulaitis G. Filament formation activates protease and ring nuclease activities of CRISPR SAVED-Lon. 2024; bioRxiv doi:08 May 2024, preprint: not peer reviewed 10.1101/2024.05.08.593097. 39362215

[21] Athukoralage J.S. , McMahon S.A., Zhang C., Grüschow S., Graham S., Krupovic M., Whitaker R.J., Gloster T.M., White M.F. An anti-CRISPR viral ring nuclease subverts type III CRISPR immunity. Nature. 2020; 577:572–575. 31942067 10.1038/s41586-019-1909-5 PMC6986909

[22] Athukoralage J.S. , Rouillon C., Graham S., Grüschow S., White M.F. Ring nucleases deactivate type III CRISPR ribonucleases by degrading cyclic oligoadenylate. Nature. 2018; 562:277–280. 30232454 10.1038/s41586-018-0557-5 PMC6219705

[23] Athukoralage J.S. , Graham S., Grüschow S., Rouillon C., White M.F. A type III CRISPR ancillary ribonuclease degrades its cyclic oligoadenylate activator. J. Mol. Biol. 2019; 431:2894–2899. 31071326 10.1016/j.jmb.2019.04.041 PMC6599890

[24] Molina R. , Garcia-Martin R., López-Méndez B., Jensen A.L.G., Ciges-Tomas J.R., Marchena-Hurtado J., Stella S., Montoya G. Molecular basis of cyclic tetra-oligoadenylate processing by small standalone CRISPR-Cas ring nucleases. Nucleic Acids Res. 2022; 50:11199–11213. 36271789 10.1093/nar/gkac923 PMC9638899

[25] Molina R. , Jensen A.L.G., Marchena-Hurtado J., López-Méndez B., Stella S., Montoya G. Structural basis of cyclic oligoadenylate degradation by ancillary type III CRISPR-Cas ring nucleases. Nucleic Acids Res. 2021; 49:12577–12590. 34850143 10.1093/nar/gkab1130 PMC8643638

[26] Rouillon C. , Athukoralage J.S., Graham S., Grüschow S., White M.F. Investigation of the cyclic oligoadenylate signaling pathway of type III CRISPR systems. Methods Enzymol. 2019; 616:191–218. 30691643 10.1016/bs.mie.2018.10.020

[27] Jo S. , Kim T., Iyer V.G., Im W. CHARMM-GUI: a web-based graphical user interface for CHARMM. J. Comput. Chem. 2008; 29:1859–1865. 18351591 10.1002/jcc.20945

[28] Berendsen H.J.C. GROMACS: a message-passing parallel molecular dynamics implementation. Comput. Phys. Commun. 1995; 91:43–56.

[29] Abramson J. , Adler J., Dunger J., Evans R., Green T., Pritzel A., Ronneberger O., Willmore L., Ballard A.J., Bambrick J. et al. Accurate structure prediction of biomolecular interactions with AlphaFold 3. Nature. 2024; 630:493–500. 38718835 10.1038/s41586-024-07487-w PMC11168924

[30] Lowey B. , Whiteley A.T., Keszei A.F.A., Morehouse B.R., Mathews I.T., Antine S.P., Cabrera V.J., Kashin D., Niemann P., Jain M. et al. CBASS immunity uses CARF-related effectors to sense 3’-5’- and 2’-5’-linked cyclic oligonucleotide signals and protect bacteria from phage infection. Cell. 2020; 182:38–49. 32544385 10.1016/j.cell.2020.05.019 PMC7728545

[31] Smalakyte D. , Kazlauskiene M., Havelund J.F., Rukšėnaitė A., Rimaite A., Tamulaitiene G., Færgeman N.J., Tamulaitis G., Siksnys V. Type III-A CRISPR-associated protein Csm6 degrades cyclic hexa-adenylate activator using both CARF and HEPN domains. Nucleic Acids Res. 2020; 48:9204–9217. 32766806 10.1093/nar/gkaa634 PMC7498309

[32] Jia N. , Jones R., Yang G., Ouerfelli O., Patel D.J. CRISPR-Cas III-A Csm6 CARF domain is a ring nuclease triggering stepwise cA4 cleavage with ApA>p formation terminating RNase activity. Mol. Cell. 2019; 75:944–956. 31326273 10.1016/j.molcel.2019.06.014 PMC6731128

[33] Hogrel G. , Guild A., Graham S., Rickman H., Grüschow S., Bertrand Q., Spagnolo L., White M.F. Cyclic nucleotide-induced helical structure activates a TIR immune effector. Nature. 2022; 608:808–812. 35948638 10.1038/s41586-022-05070-9

[34] Du L. , Zhang D., Luo Z., Lin Z. Molecular basis of stepwise cyclic tetra-adenylate cleavage by the type III CRISPR ring nuclease Crn1/Sso2081. Nucleic Acids Res. 2023; 51:2485–2495. 36807980 10.1093/nar/gkad101 PMC10018336

[35] Athukoralage J.S. , McQuarrie S., Grüschow S., Graham S., Gloster T.M., White M.F. Tetramerisation of the CRISPR ring nuclease Crn3/Csx3 facilitates cyclic oligoadenylate cleavage. eLife. 2020; 9:e57627. 32597755 10.7554/eLife.57627 PMC7371418

