# Supplementary material for: The SAVED domain of the type III CRISPR protease CalpL is a ring nuclease
Source: Nucleic Acids Res. 2024 Aug 21;52(17):10520–32. doi: 10.1093/nar/gkae676 (PMC11417357; doi:10.1093/nar/gkae676)
Supplement: gkae676_Supplemental_Files [file gkae676_supplemental_files.zip › 2024-07-18 CalpL revision_final_SI.pdf]

Supplementary Information

The SAVED domain of the type III CRISPR protease CalpL is a ring nuclease

Sophie C. Binder[1,#], Niels Schneberger[1,#], Maximilian Schmitz[1], Marianne Engeser[2], Matthias Geyer[1], Christophe Rouillon[3], Gregor Hagelueken[1,*]

[1] Institute of Structural Biology, Venusberg-Campus 1, University of Bonn

[2] Kekulé Institute of Organic Chemistry and Biochemistry, University of Bonn

[3] Institut Pasteur, Université Paris Cité, Synthetic Biology, 75015 Paris, France

[#]equal contribution

[*]hagelueken@uni-bonn.de

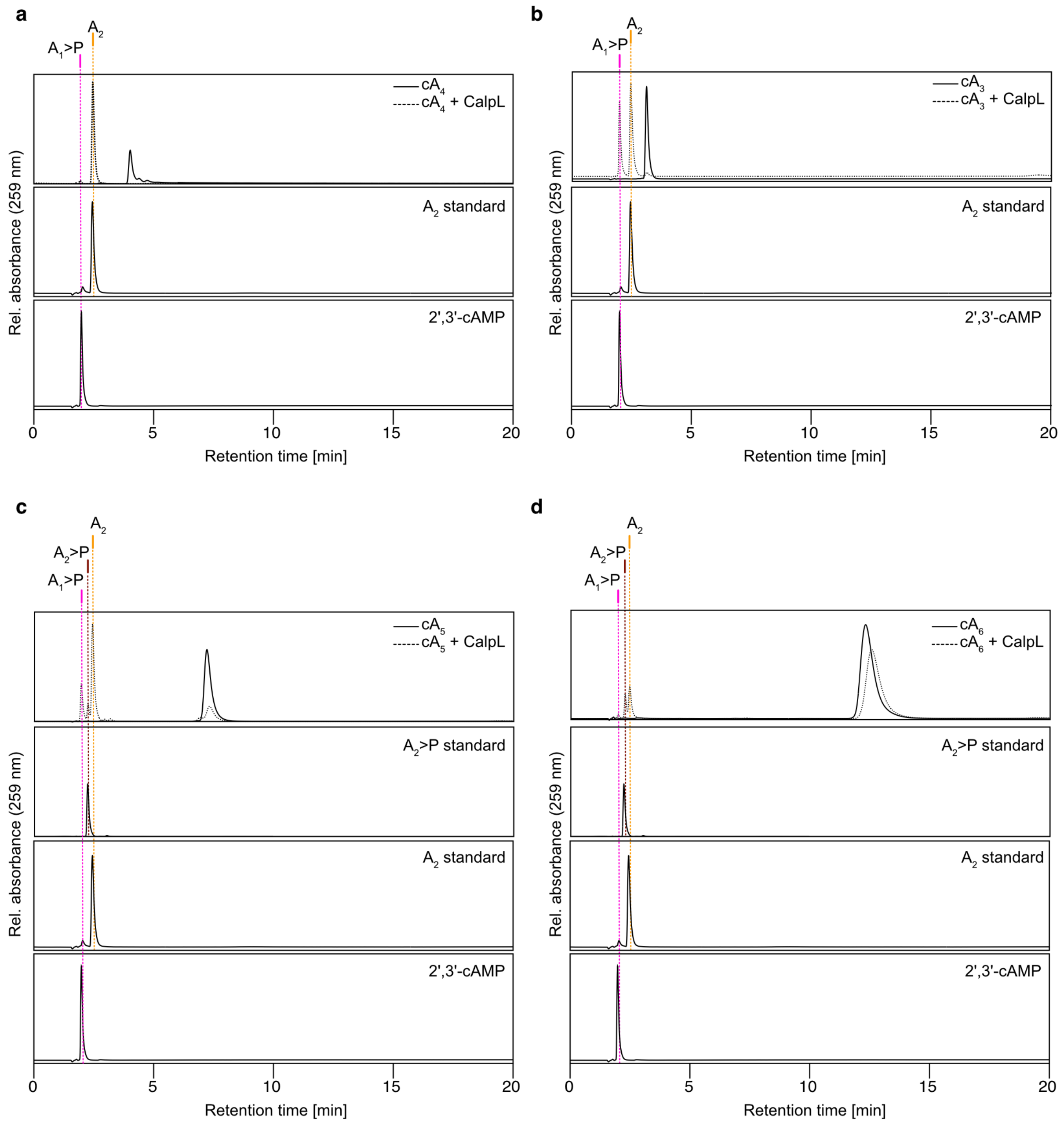

Fig. S1 | Identification of cOA degradation products by comparison to HPLC oligoadenylate standards. a-d) HPLC traces recorded at 259 nm showing the result of incubating different cOAs with (dashed lines) and without (solid lines) CalpL for 120 min at 60 °C. The A_2>P standard was generated by incubating MazF with the respective substrate for 60 min at 37 °C. HPLC traces are representative of three replicates.

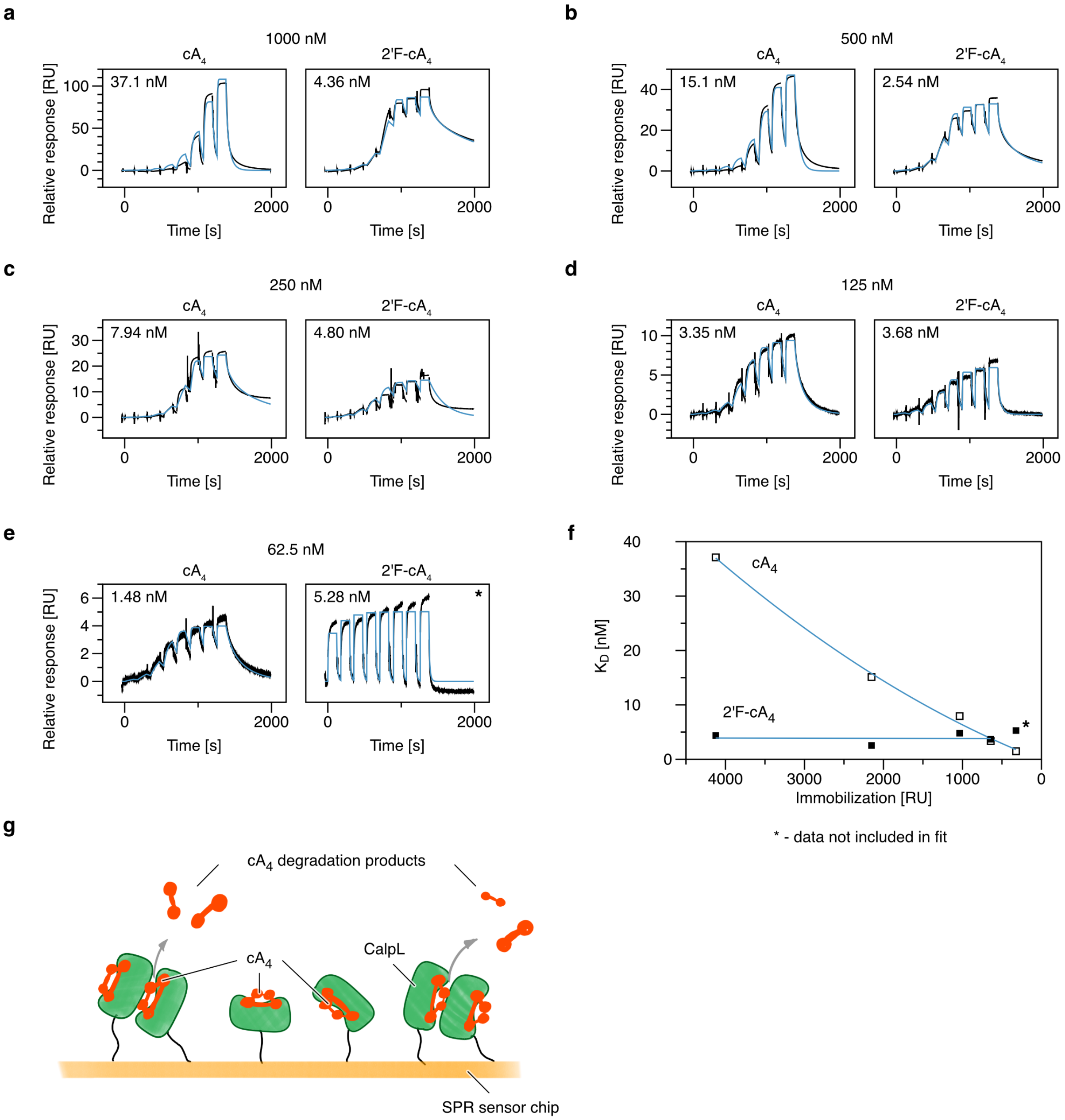

Fig. S2 | SPR titration experiment illustrating the correlation between apparent K_D values and CalpL immobilization rates. a-e) Surface plasmon resonance (SPR) sensorgrams of cA_4 and 2'F-cA_4 binding measurements using different immobilization rates of wildtype CalpL (final concentrations used for the immobilization process are indicated; a) 1000 nM; b) 500 nM; c) 250 nM; d) 125 nM; e) 62.5 nM). Binding parameters were determined by applying a 1:1 interaction model. Resultant dissociation constants (K_D) are indicated in each panel. For all measurements, the association time was set to 160 s. **f)** Diagram depicting the relationship of the apparent K_D value of cA_4 and 2'F-cA_4 binding determined in a-e) plotted against the CalpL immobilization rate (RU). **g)** Cartoon illustrating CalpL dimerization on the SPR sensor chip surface resulting in ring nuclease activity of CalpL and cA_4 cleavage during the measurement.

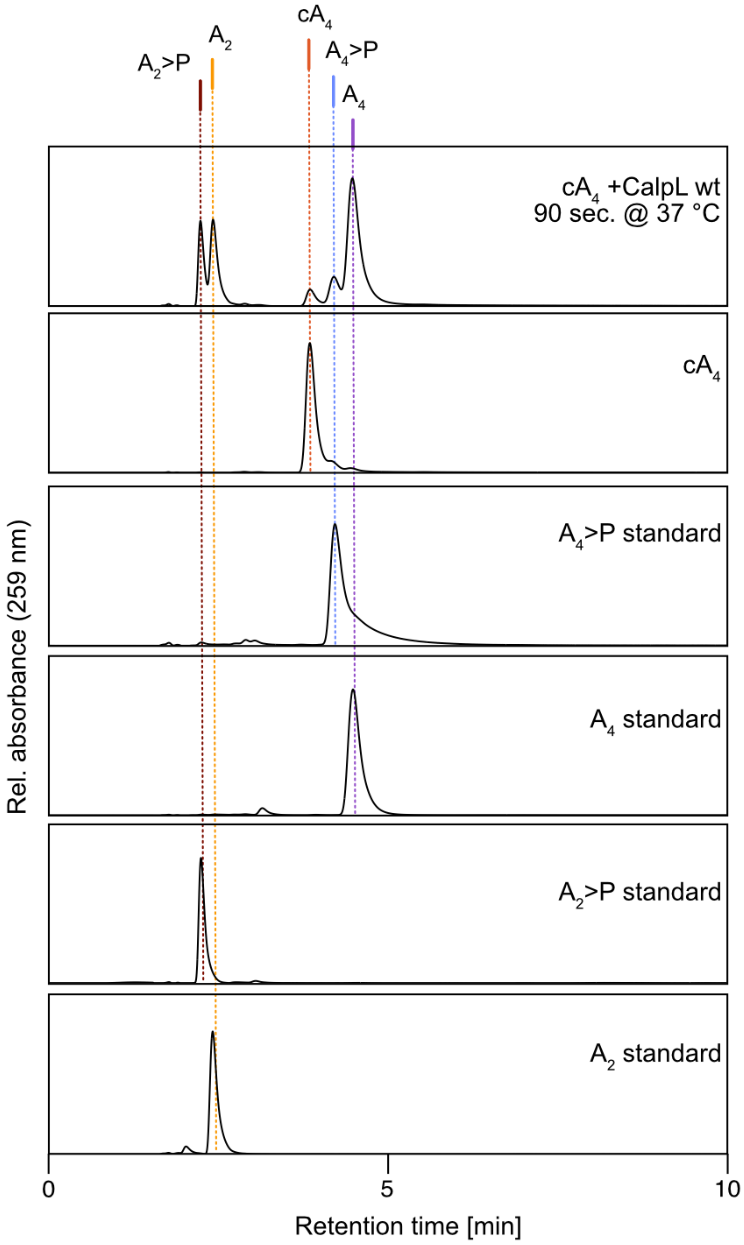

Fig. S3 | Identification of cA_4 degradation products by comparison to HPLC oligoadenylate standards. HPLC traces recorded at 259 nm showing the reaction intermediates and product generated upon incubation of 30 µM cA_4 with 3 µM CalpL at 37 °C for 90 sec. Standards containing cyclic phosphate groups were generated by incubating MazF with the respective substrate for 60 min at 37 °C. HPLC traces are representative of three replicates.

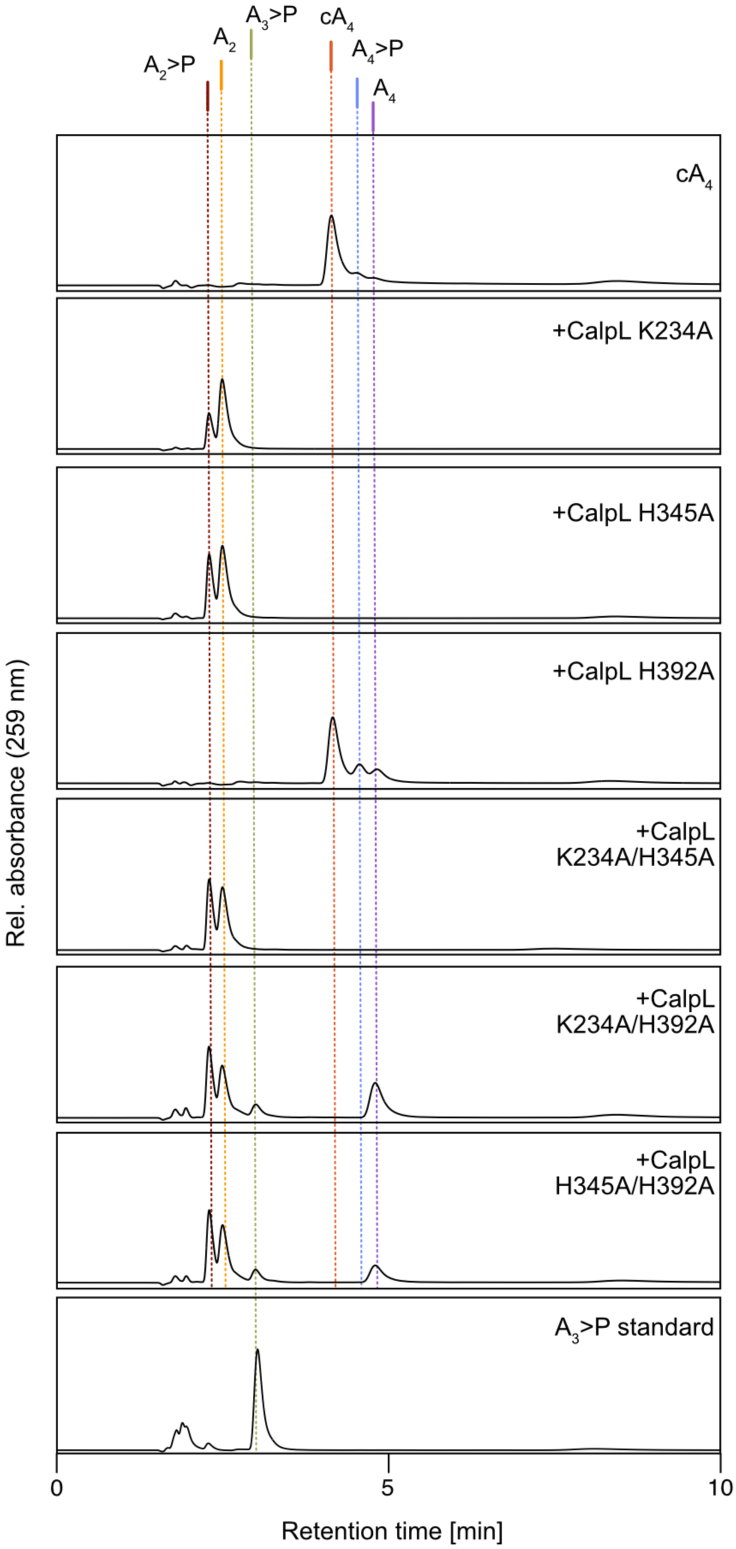

Fig. S4 | Nuclease reaction endpoint experiments of SAVED topside double mutants. HPLC traces recorded at 259 nm showing the reaction intermediates and products generated upon incubation of 15 µM cA_4 with 1.5 µM of the respective CalpL single or double mutant for 120 min at 60 °C. HPLC traces are representative of three replicates. The A_3>P standard was generated by incubating MazF with the respective substrate for 60 min at 37 °C.

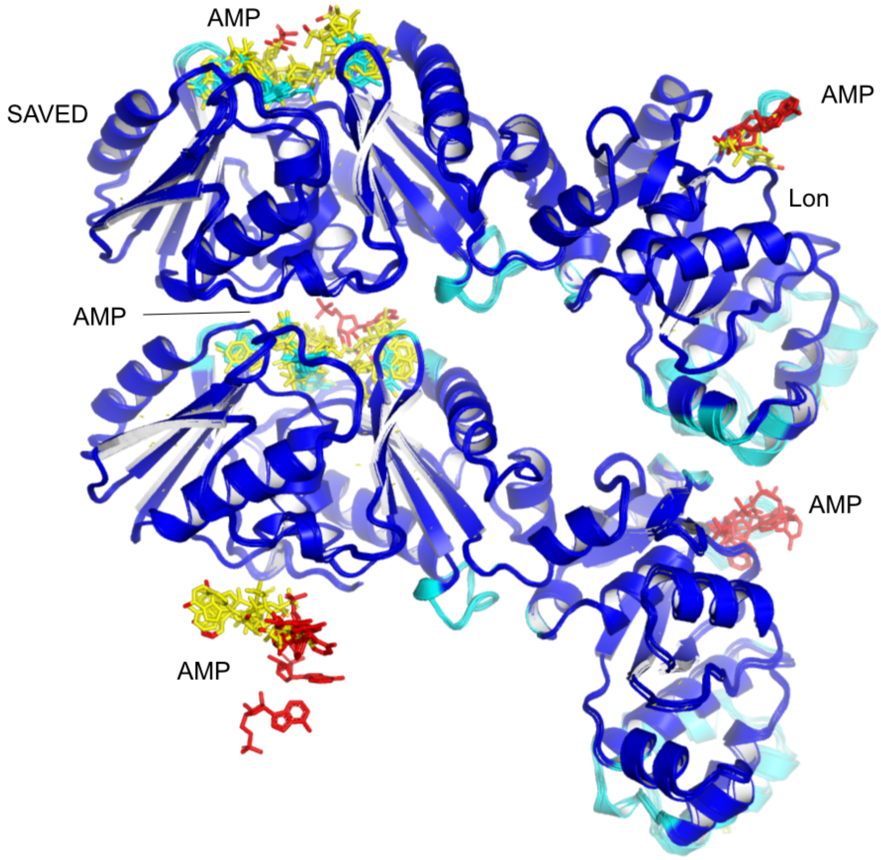

Fig. S5 | AlphaFold3 model of CalpL dimer. Five superimposed AlphaFold3 models of a CalpL dimer (cartoon) in the presence of 12 AMP ligands (sticks). The phosphate group of the AMP ligands near the Lon protease active site coincide with a sulfate ion that was co-crystallized with CalpL (PDB-id: 7qda). The color coding represents the pLDDT score of AlphaFold3 (blue: >90, cyan: 70-90, yellow: 50-70, orange: <50).

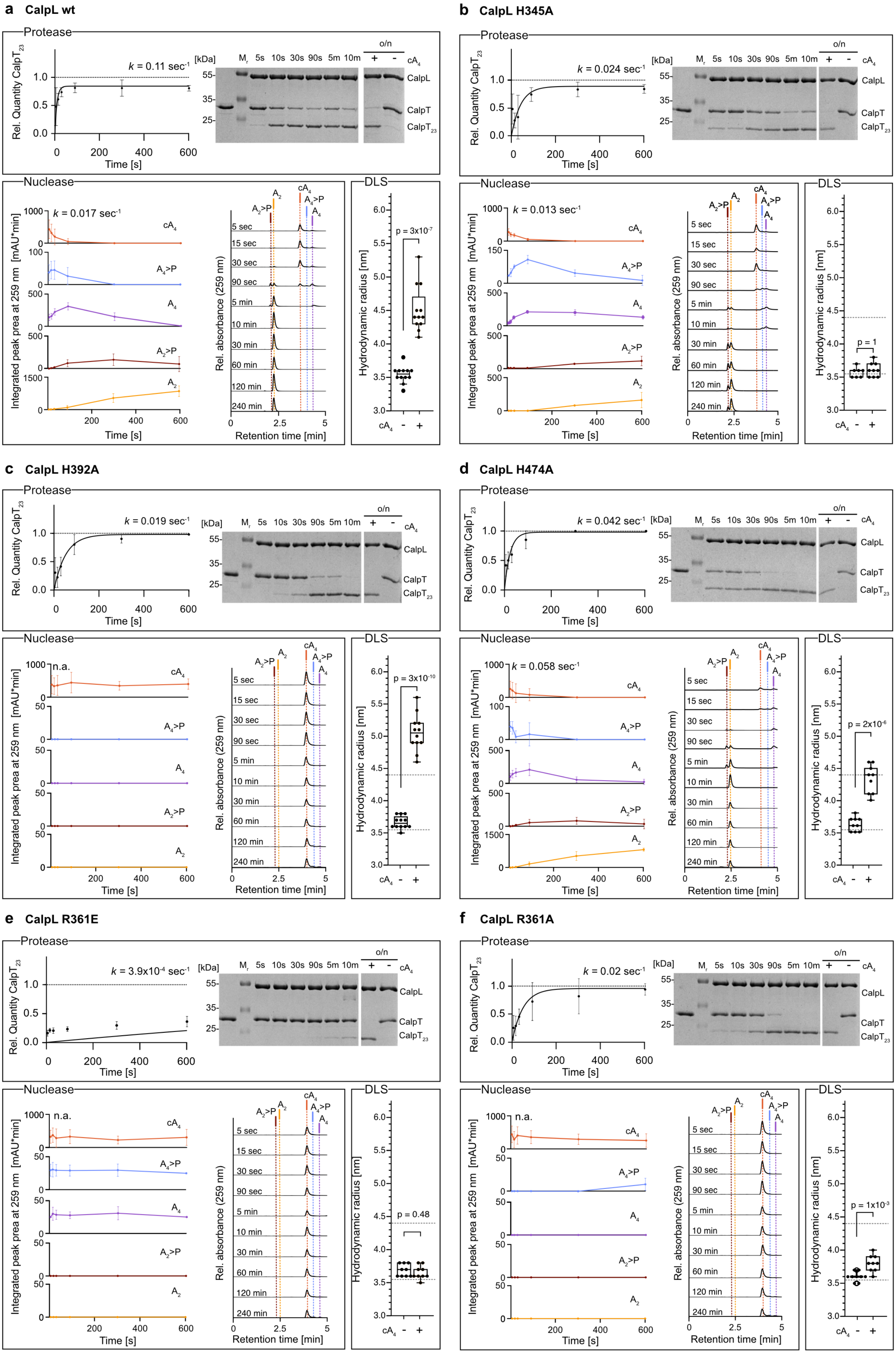
a CalpL wt
Protease
k = 0.11 sec-1
Rel. Quantity CalpT23
Time [s]
[kDa]
Mr 5s 10s 30s 90s 5m 10m
o/n
cA4
CalpL
CalpT
CalpT23
Nuclease
k = 0.017 sec-1
Integrated peak prea at 259 nm [mAU*min]
A4>P
A4
A2>P
A2
Rel. absorbance (259 nm)
Retention time [min]
DLS
p = 3x10-7
Hydrodynamic radius [nm]
b CalpL H345A
k = 0.024 sec-1
k = 0.013 sec-1
p = 1
c CalpL H392A
k = 0.019 sec-1
n.a.
p = 3x10-10
d CalpL H474A
k = 0.042 sec-1
k = 0.058 sec-1
p = 2x10-6
e CalpL R361E
k = 3.9x10-4 sec-1
p = 0.48
f CalpL R361A
k = 0.02 sec-1
p = 1x10-3
5 sec
15 sec
30 sec
90 sec
5 min
10 min
30 min
60 min
120 min
240 min

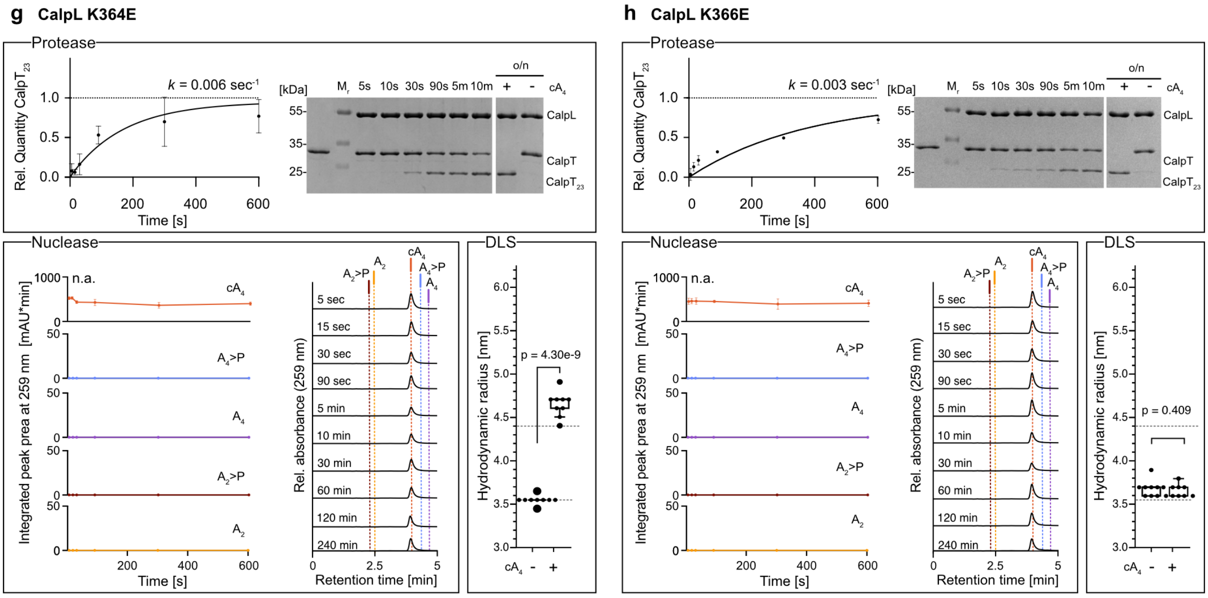

Fig. S6 | Interdependence of ring nuclease and protease activities of CalpL. Upper panels: Quantification of CalpT cleavage by incubating 3 µM of the respective CalpL mutant with 3 µM CalpT at 37 °C for the indicated time points. A Coomassie-stained 15% SDS-PAGE gel used for quantification is included for reference. Left lower panels: Quantification of nuclease reaction intermediates and products generated upon incubation of 1.5 µM CalpL and 15 µM cA_4 at 37 °C for the indicated time points. HPLC traces used for quantification by peak area integration at 259 nm are shown exemplary. Right lower panels: Hydrodynamic radii of CalpL determined at a sample concentration of 86 µM (5 mg/ml) in the presence or absence of 86 µM cA_4. Dashed lines mark the hydrodynamic radii of wildtype CalpL with (4.5 ± 0.35 nm) and without (3.5± 0.12 nm) cA_4. All experiments were performed in triplicates. Bars of DLS data display median and interquartile range; *p*-values for two-tailed t-tests are indicated.

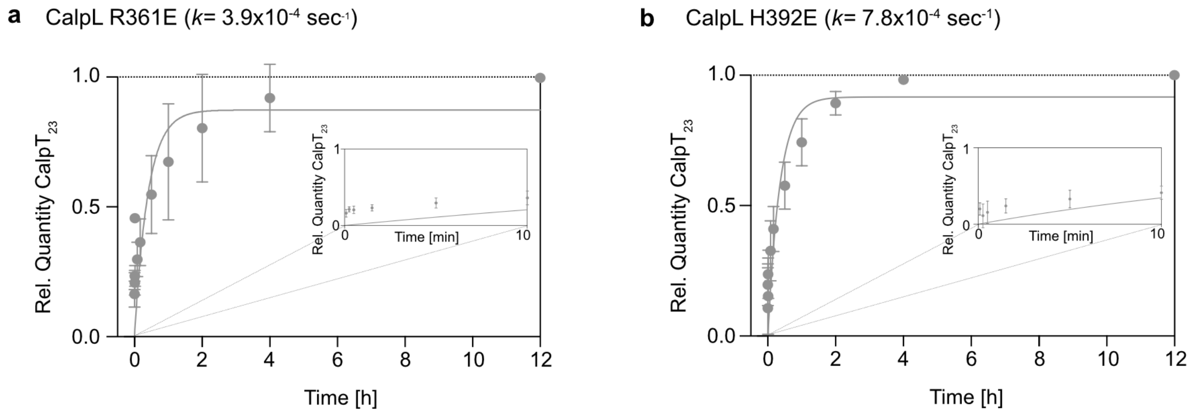

Fig. S7 | Protease time course experiments including all time points of the quantification of CalpT cleavage (supplementary to time courses shown in Fig S6). a, b) Quantification of CalpT cleavage as described in Fig. S6 depicting all time points measured during the time course (5 sec, 15 sec, 30 sec, 90 sec, 5 min, 10 min, 30 min, 60 min, 120 min, 240 min, 720 min). Rate constants *k* were calculated by fitting a one phase decay model (Y=(Y0-Plateau)*exp(-K*X)+Plateau) to all time points.

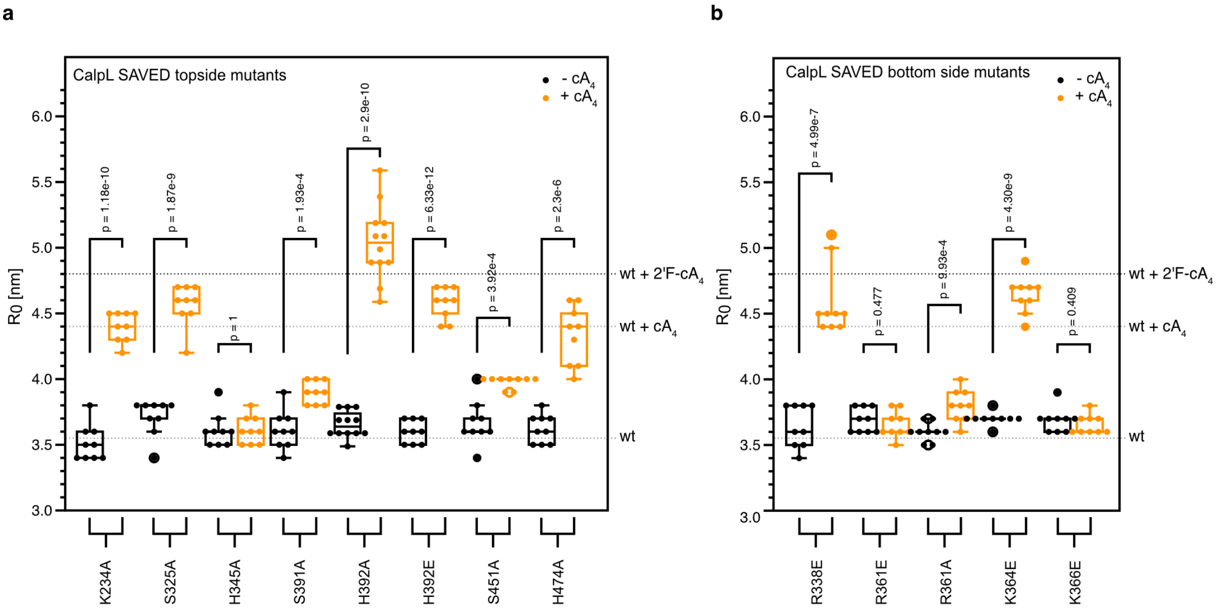

Fig. S8 | Hydrodynamic radii of SAVED topside and bottom side mutants. Hydrodynamic radii of SAVED topside **(a)** or bottom side **(b)** mutants determined in the presence (orange) and absence (black) of cA_4 by dynamic light scattering (DLS). Mean hydrodynamic radii of wildtype CalpL in the presence of cA_4 (grey traces) or 2'F-cA_4 (black traces) are indicated. Measurements were performed at a sample concentration of 86 µM (5 mg/ml) and at a temperature of 20 °C. For each condition, three measurement cycles of each 20 single data acquisitions with acquisition times of 3 sec were recorded. Bars of DLS data display median and interquartile range; *p*-values for two-tailed t-tests are indicated.

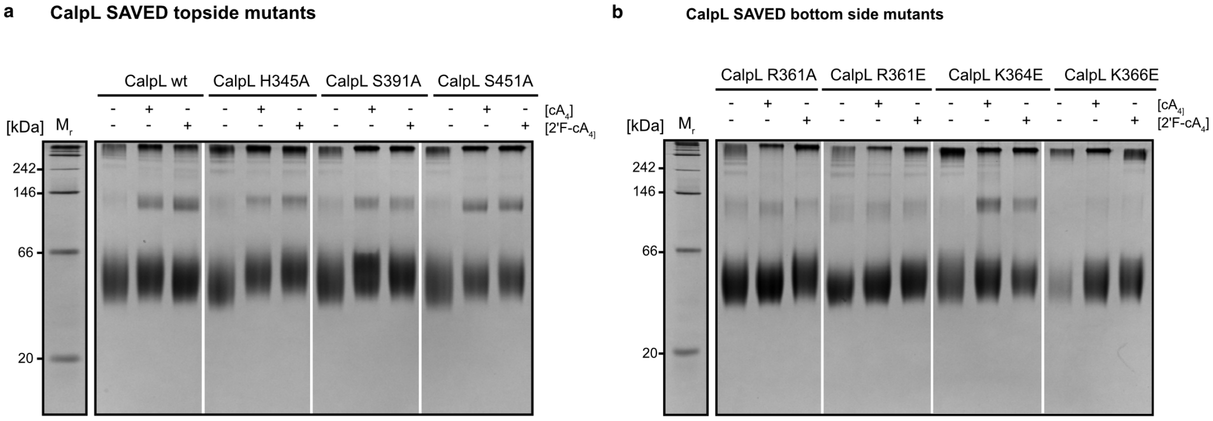

Fig. S9 | Coomassie-stained 10 % native PAGE analysis of CalpL oligomerization experiment. a) Coomassie-stained 10 % native PAGE analysis of CalpL SAVED topside mutants oligomerization experiment. For the assay, 15 µM of CalpL was incubated with (+) or without (-) 75 µM cA_4 or 2'F-cA_4 for 30 min at 37 °C in the presence of 0.5 mM bissulfosuccinimidyl suberate (BS3) crosslinking agent. **b)** as in a) but with SAVED bottom side mutants.

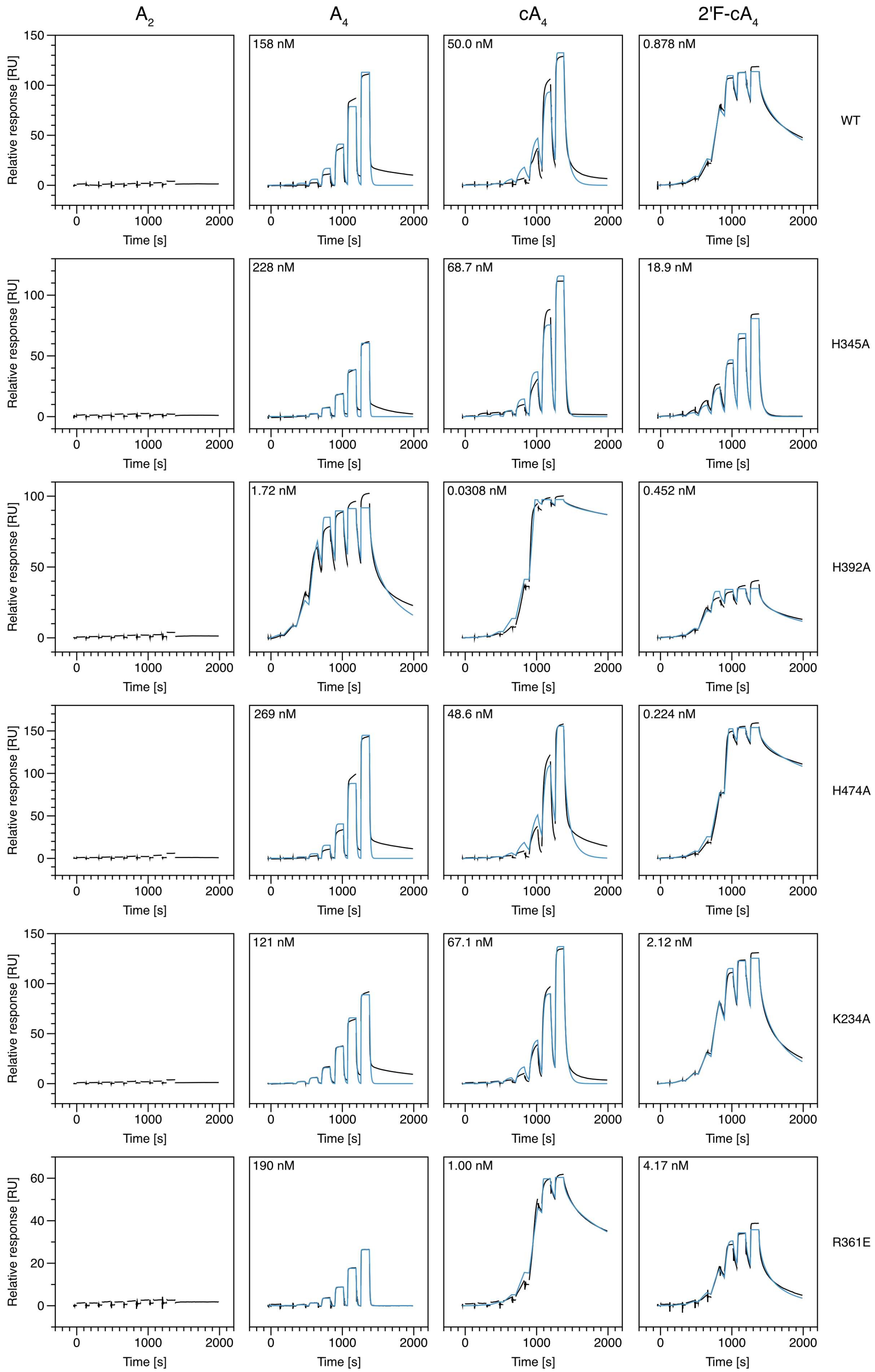
A2
A4
cA4
2'F-cA4
158 nM
50.0 nM
0.878 nM
WT
228 nM
68.7 nM
18.9 nM
H345A
1.72 nM
0.0308 nM
0.452 nM
H392A
269 nM
48.6 nM
0.224 nM
H474A
121 nM
67.1 nM
2.12 nM
K234A
190 nM
1.00 nM
4.17 nM
R361E
Relative response [RU]
Time [s]

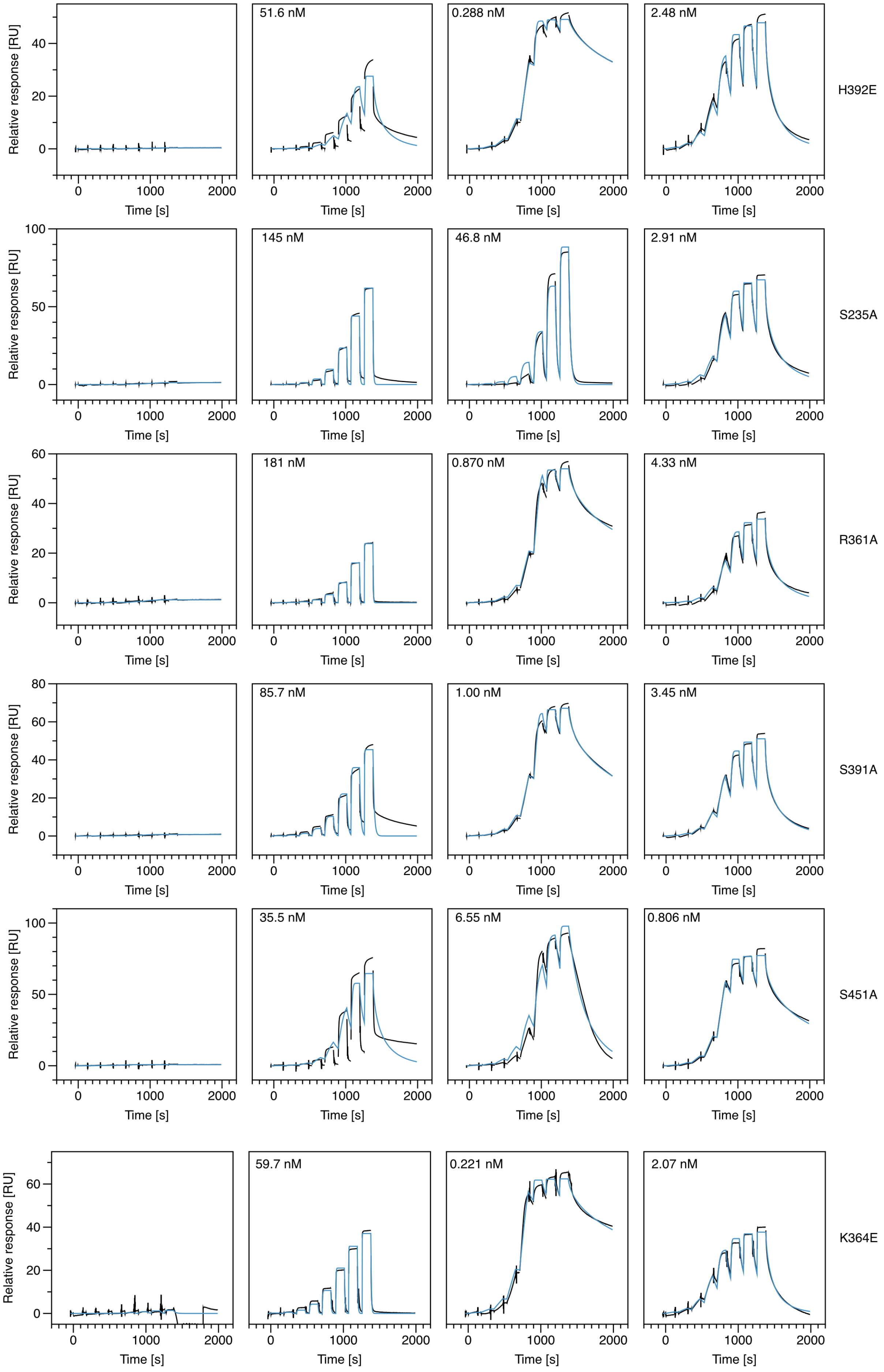

Relative response [RU]
Time [s]
51.6 nM
0.288 nM
2.48 nM
H392E
145 nM
46.8 nM
2.91 nM
S235A
181 nM
0.870 nM
4.33 nM
R361A
85.7 nM
1.00 nM
3.45 nM
S391A
35.5 nM
6.55 nM
0.806 nM
S451A
59.7 nM
0.221 nM
2.07 nM
K364E

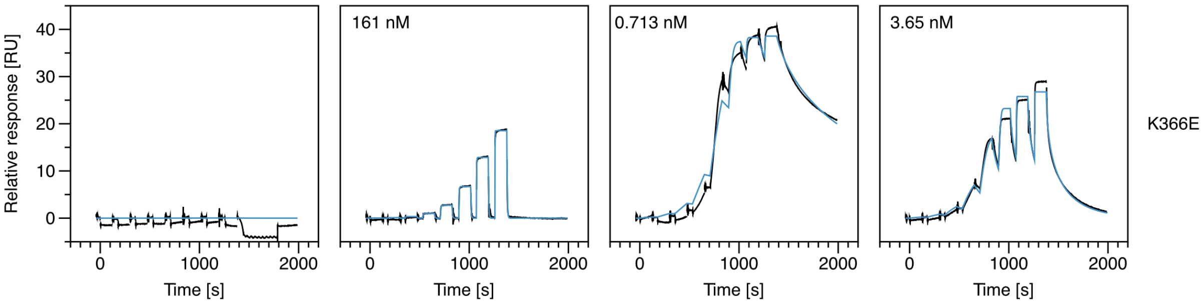

Fig. S10 | Binding kinetics and affinity measurements of 2'F-cA_4, cA_4, A_4, and A_2 towards different CalpL variants. Surface plasmon resonance (SPR) measurements were performed by immobilizing the respective CalpL variants using amine-coupling to a CM5 sensor chip. For A_4 and A_2, single-cycle kinetics were determined by injections of increasing concentrations of analyte (0.26 nM, 0.78 nM, 2.33 nM, 7 nM, 21 nM, 63 nM, 189 nM, and 567 nM). For cA_4 and 2'F-cA_4, a lower concentration range was used (0.086 nM, 0.26 nM, 0.78 nM, 2.33 nM, 7 nM, 21 nM, 63 nM, and 189 nM). For all measurements, the association and dissociation time was set to 120 s and 600 s, respectively. Binding parameters were determined by applying a 1:1 interaction model. Resultant dissociation constants (K_Ds) are indicated in each panel. As explained in the main text, the K_D of cA_4 and A_4 are affected by the nuclease reaction and are therefore "apparent" K_Ds. The immobilization rates of CalpL were slightly different for the various measurements. Hence, as explained in the main text, this will affect the apparent K_Ds of the cA_4 and A_4 measurements.

Table S1. Accurate mass determination from sample fractions collected after HPLC separation.

	signal assignment	measured *m/z*	calculated *m/z*	elemental composition
A_4	$[M\text{-}2H]^{2-}$	666.1032	666.1030	$[C_{40}H_{48}N_{20}O_{25}P_4]^{2-}$
	$[M\text{-}3H\text{+}Na]^{2-}$	677.0893	677.0940	$[C_{40}H_{47}N_{20}O_{25}P_4Na]^{2-}$
A_4>P	$[M\text{-}2H]^{2-}$	657.0974	657.0978	$[C_{40}H_{46}N_{20}O_{24}P_4]^{2-}$
	$[M\text{-}3H\text{+}Na]^{2-}$	668.0843	668.0887	$[C_{40}H_{45}N_{20}O_{24}P_4Na]^{2-}$
cA_4	$[M\text{-}2H]^{2-}$	657.0951	657.0978	$[C_{40}H_{46}N_{20}O_{24}P_4]^{2-}$
	$[M\text{-}3H\text{+}Na]^{2-}$	668.0885	668.0887	$[C_{40}H_{45}N_{20}O_{24}P_4Na]^{2-}$
A_2	$[M\text{-}H]^{-}$	675.1096	675.1083	$[C_{20}H_{25}N_{10}O_{13}P_2]^{-}$
	$[M\text{-}2H\text{+}Na]^{-}$	697.0932	697.0903	$[C_{20}H_{24}N_{10}O_{13}P_2Na]^{-}$
A_2>P	$[M\text{-}H]^{-}$	657.1007	657.0978	$[C_{20}H_{23}N_{10}O_{12}P_2]^{-}$
	$[M\text{-}2H\text{+}Na]^{-}$	679.0869	679.0797	$[C_{20}H_{22}N_{10}O_{12}P_2Na]^{-}$

Table S2. Collection of ring nuclease rates, protease rates, and hydrodynamic radii of all CalpL variants. All values represent mean ± SD and are representative of three replicates. Nuclease rates of mutants lacking ring nuclease activity are labeled as n.a. (not applicable). Dash (-) indicates values which were not determined in our experiments.

CalpL variant	Nuclease rate *k* [sec^{-1}]	Protease rate *k* [sec^{-1}]	Hydrodynamic radius [nm] - cA_4	Hydrodynamic radius [nm] + cA_4
wildtype + cA_4	0.017 ± 0.0030	0.110 ± 0.0216	3.54 ± 0.124	4.52 ± 0.346
wildtype + 2'F-cA_4	n.a.	0.016 ± 0.0027	3.54 ± 0.124	4.89 ± 0.209
H345A	0.013 ± 0.0028	0.024 ± 0.0064	3.61 ± 0.127	3.61 ± 0.105
H392A	n.a.	0.019 ± 0.0032	3.67 ± 0.099	5.05 ± 0.281
H392E	-	7.88e-004 ± 2.55e-004	3.60 ± 0.087	4.58 ± 0.120
H474A	0.058 ± 0.0192	0.042 ± 0.0079	3.62 ± 0.109	4.33 ± 0.223
R361A	n.a.	0.020 ± 0.0036	3.60 ± 0.071	3.80 ± 0.122
R361E	n.a.	3.96e-004 ± 3.343e-005	3.69 ± 0.093	3.66 ± 0.101
K364E	n.a.	0.006 ± 0.0012	3.70 ± 0.050	4.64 ± 0.142
K366E	n.a.	0.003 ± 0.0004	3.69 ± 0.093	3.66 ± 0.073
R361E + H392E	-	0.008 ± 0.0022	-	-

Table S3. Overview of kinetic and steady-state affinities of 2'F-cA_4, cA_4, and A_4 (with a cyclic 3' phosphate) towards the different CalpL variants.

CalpL variant	Affinity 2'F-cA_4 [nM]<br>Kinetic/ steady-state	Affinity cA_4 [nM]<br>Kinetic/ steady-state	Affinity A_4 [nM]<br>Kinetic/ steady-state
wildtype	0.878/ 5.28	50/ 55.8	158/ 146
K234A	2.12/ 5.29	67.1/ 67.4	121/ 129
S235A	2.91/ 4.98	46.8/ 41.0	145/ 135
H345A	18.9/ 22.6	68.7/ 60.6	228/ 219
S391A	3.45/ 5.66	1.00/ 8.61	85.7/ 105
H392A	0.452/ 2.31	0.0308/ 9.61	1.72/ 4.19
H392E	2.48/ 3.82	0.288/ 5.29	51.6/ n.a.
S451A	0.806/ 4.40	6.55/ 11.9	35.5/ 83.6
H474A	0.224/ 7.88	48.6/ 68.5	269/ 237
R361A	4.33/ 7.87	0.87/ 10.8	181/ 181
R361E	4.17/ 9.34	1.00/ 15.3	190/ 190
K364E	2.07/ 2.78	0.221/ 3.50	59.7/ 66.9
K366E	3.65/ 5.80	0.713/ 5.18	161/ 149
