# Supplementary material for: The SAVED domain of the type III CRISPR protease CalpL is a ring nuclease
Source: Nucleic Acids Res. 2024 Aug 21;52(17):10520–32. doi: 10.1093/nar/gkae676 (PMC11417357; doi:10.1093/nar/gkae676)
Supplement: gkae676_Supplemental_Files [file gkae676_supplemental_files.zip › sequence_alignment.pdf]

```
tr|A0A4R1GDP6|A0A4R1GDP6_9BACT    1  ..MSYLLRLLEKEK....FSTVLSYLEGWK.RGEVEL..EK..EEI....AT..AVKCKR
tr|A0A1E3G168|A0A1E3G168_9BACT    1  ..MAFVNVLR.........MNVQTYIELLR.NGVITI..EEAVKDLEML.DE..DERDIK
tr|A0A7C5U2L3|A0A7C5U2L3_9BACT    1  ..MSFVDFAE.........LYSDEFIKLYE.NGLITP..LEFVKTFKSL.PS..SYFQIK
tr|A0A285P190|A0A285P190_9AQUI    1  ..MLYSFSSF..........KEEDLLGFLH.AGEL....DEHIADV....FK..EFNELS
tr|A0A6C1BRW5|A0A6C1BRW5_9AQUI    1  ...MWNFSLF..........KEEDLIEFLH.RGDL....EEVLVDL....IR..QWDYLS
tr|A0A3M1C218|A0A3M1C218_9BACT       ............................................................
tr|A0A5P1V5W6|A0A5P1V5W6_9BACT    1  ...MFRFEVF..........EEDHLLKFLW.QGLL....DEYIEDL....FL..RWDLFS
tr|A0A1M6Q5V1|A0A1M6Q5V1_9AQUI    1  MSKRWNYEEI..........RDAHFKVLLE.EGKL....DDKLTEI....FE..DFWTLS
tr|A0A7V3ZWQ1|A0A7V3ZWQ1_UNCW3    1  ...MFRFCEF..........EVSHFTKLLR.AGKI....NDFIEDI....LE..QFQELK
tr|D3SNA5|D3SNA5_THEAH            1  ...MWSFSEI..........EERHFEVLLW.SGKM....DDLLDEV....MR..EFYSLP
tr|A8UTR1|A8UTR1_9AQUI            1  ...MFRFSEI..........EDRHFKGFLW.SGKL....DEYMDEV....FE..QLWELS
tr|W0DEP5|W0DEP5_9AQUI            1  ...MWKFWEI..........ELKHFKTLLE.SGKL....DDHIEGL....YS..QFWELP
tr|A0A7X7TR99|A0A7X7TR99_9THEM    1  ...MFRWLLE.........EDRKKYVSFLE.TGAISL..DEFIDDLISV.RK..DNASKY
tr|A0A841GNB0|A0A841GNB0_9BACT    1  ...MFSRLLS.........SNSSDFLKILE.RDDITV..KEAIESFIN..AE..DFDNKF
tr|K2NM55|K2NM55_THEA7               ............................................................
tr|A0A7V4KD75|A0A7V4KD75_FERPE    1  ..MAYSNLLK.........NDVLKYLDFVK.KGEISL..GEIPVDLSRL.DK..HIPQRK
tr|A7HMU9|A7HMU9_FERNB            1  ..MSFSNVMA.........NDVKKYLEFVS.AGEIEI..GMVLHDFSLL.SP..NIPNYQ
tr|A0A101EV44|A0A101EV44_9THEM    1  .........M.........RDSAEYFRLVQ.SGVLSK..EKLLRDFELV.PK..DHPDRS
tr|A0A432Q0P4|A0A432Q0P4_9AQUI    1  ..MKI.............PQKTSFISNYLNRPSQLS...PEELQVILEE.YPYKDFS.YE
tr|A0A1V5WQ44|A0A1V5WQ44_9BACT    1  ..MTF.PELVTAYLKEGTELLKNKILDYLN.TNNFSEE..DWLPI.IDLLFN.......P
tr|B0VHB4|B0VHB4_CLOAI            1  .MPKF.NETADKYLKSGSAEAELIILQYIQ.QDRVSEDDEEWVYNLLEKANN.......P
tr|A0A238XV58|A0A238XV58_9BACT    1  ..........MKFD....EYPVDIVIEFVR.EGRTIP..FEENEEYIREIFLGESVT.D.
tr|C1DV83|C1DV83_SULAA            1  ...MVKQFL..EDL....ERFYPVILNLIK.DGNIK...KEWLADLYSI.FNMEDS.PYI
tr|B2V8L9|B2V8L9_SULSY            1  ..MHIKQLL..K......NKRFEVIKALVE.SKKIK...QEWLEDLYSI.LLKQDTDVEI
tr|C4FJQ9|C4FJQ9_9AQUI               ............................................................
tr|F8E961|F8E961_FLESM            1  .MKTIFDNI..Q.E....GRNHKIIEQCIK.DGSIN...QDSLPYCYSL.FSKTEFA.EY
tr|E4TFD9|E4TFD9_CALNY            1  .MSPL.SKY..F.E....PEKYKILLNSIR.DGNFLP..GLELEDYYRA.FSDETFR.DE
tr|A0A1R1MKQ3|A0A1R1MKQ3_9BACT    1  ..MGWFRKLITKEI....KLTTEEINKLIR.SGEVD...GEDLPFLLKA.IKEKR...LP
tr|A0A238Y624|A0A238Y624_9BACT    1  ..MSWVDLY..RDG....KLREDEALKLVE.SGEIG...EKDLSELLIL.SKEKK...LP
tr|A0A285NI51|A0A285NI51_9AQUI    1  ..MSLDDLL..AYY....GY..DFLKSKII.DGKIE...KDALVSYYQI.FNSEY...D.
tr|A0A432RBX1|A0A432RBX1_9AQUI    1  .MLRLKVIK..QLY....QTDKNLAIRLIN.EEEIK...PEETQELYEL.LNEKD...D.
tr|A0A2N7Q1Y2|A0A2N7Q1Y2_9AQUI       ............................................................
```

```
tr|A0A4R1GDP6|A0A4R1GDP6_9BACT   44  PEVSFKAVEGLFERFSFEEVKEIFK..........ELFNITPL...SPPVKFSFPAINEK
tr|A0A1E3G168|A0A1E3G168_9BACT   44  LRLQYGIVEVLMRGGALDS..SILSK...VLQLP.DDFGTLKL...LGSYKFEFPVVITR
tr|A0A7C5U2L3|A0A7C5U2L3_9BACT   44  VRMIFKFIKMFVEKGLGDK..EILES...ILGDR.ELAETIAS...TEPYQFDFPVVLPE
tr|A0A285P190|A0A285P190_9AQUI   38  PFVQFELIKYVKEKSVY.VDVHVLSK...TLGTSQKTAEEILK...GEFKIFTFPAVSTQ
tr|A0A6C1BRW5|A0A6C1BRW5_9AQUI   37  PGVHFALVKYLRLSERY.FDEEKLAK...ALGIKKAVAKALLE...NPYVEFEFPAVSER
tr|A0A3M1C218|A0A3M1C218_9BACT    1  .................................................MEFEFPAVSDG
tr|A0A5P1V5W6|A0A5P1V5W6_9BACT   37  PKIQFELIHYVKTRLKDVISPKIIAR...ALNIKNSDAQKILT...AKEKIFEVLLVEKE
tr|A0A1M6Q5V1|A0A1M6Q5V1_9AQUI   40  PFKQYELISYIKKHGKR.PEWKVLKK...VLGLSEDDTHKFLI...GDPIEVKFPVVDLT
tr|A0A7V3ZWQ1|A0A7V3ZWQ1_UNCW3   37  PEAQYEVVKYLREKRVP.VSLDVFAK...ALGIKQEDARKILM...EGARVVPLALVSS.
tr|D3SNA5|D3SNA5_THEAH           37  AKSQFNLVWYIKEKRVH.PSVNLMAE...VWEVPEEDVEAWLN...GPYRLFLVPVVDEK
tr|A8UTR1|A8UTR1_9AQUI           37  SFGQFELVRYVKEKGAN.PSLGVLSS...TFGIDKEDAKRFLR...NPYREFFVPVVGE.
tr|W0DEP5|W0DEP5_9AQUI           37  PSHQYELVKYSKEKEVF.PSIQTFRK...VFKVSEETAVKFFK...EKHITFEFPVVSSD
tr|A0A7X7TR99|A0A7X7TR99_9THEM   43  PEMVFLAIRKAISEKYIDV..D...KFSTLLNVRSECVLELLR...AEYKVVKFPVVSKD
tr|A0A841GNB0|A0A841GNB0_9BACT   42  .LVFYEIVKKIKEKKIVDLNQELLKKFFN...GNEEIIFDIYK...NDFVEVFFPIVNKL
tr|K2NM55|K2NM55_THEA7               ............................................................
tr|A0A7V4KD75|A0A7V4KD75_FERPE   44  .LIVYEILKFYLLQNYKEQAINLLITEYSSLGLKKEEAYLLAW...SKFVEVKFPVVEAD
tr|A7HMU9|A7HMU9_FERNB           44  .KIFFEILRYLLKKGLEEVAARLFSIASK...LSLSDSYRIVS...SEYFEAKFPIVYNS
tr|A0A101EV44|A0A101EV44_9THEM   37  .QIFYKLFKLLLQDNDRSFAVLLLSREYS...LSFELARRIVS...SDYREVKFPVVEGE
tr|A0A432Q0P4|A0A432Q0P4_9AQUI   41  ENINYKVIKLLRSVAIS.IKAEKLSE...KLNISKQTAKEILT...NQAVEVKFPVVIEG
tr|A0A1V5WQ44|A0A1V5WQ44_9BACT   47  YSNKVSAFAWLALIAKS.HQDSDLAK...RLDLNPGQFAEVFQ...SRIRKAYFPIIDTQ
tr|B0VHB4|B0VHB4_CLOAI           51  Y.IKLNALLWLSAKR...KYLTQLSK...LWGISENELKSLSQ...QQPKIGLFPAVDSR
tr|A0A238XV58|A0A238XV58_9BACT   42  ............KHFFL.QNLLLFRL...LDEIPISEIEKVMAMEIVDVKHFEFPAISVS
tr|C1DV83|C1DV83_SULAA           46  TPLKFHLYSLLVHHPEF.LDYKLLSE...KLNLSEDEIYEIFV.KGKQAFEVYFPVYLPD
tr|B2V8L9|B2V8L9_SULSY           46  TQAKYEIIKLLLTEKKY.LNFELLTK...TLNLDQQTAIEIMR...NPFKEVYFPTYNIE
tr|C4FJQ9|C4FJQ9_9AQUI               ............................................................
tr|F8E961|F8E961_FLESM           47  RSLKSNFLQSVFIYDHL.FNVEYLCE...HLQITENDCYALKN...AKIKRAYFPVTSHN
tr|E4TFD9|E4TFD9_CALNY           47  EYLKSTLFLAIRRYSKF.QNLKLLSN...HFKMDEDNLRVLLN...EDYKIASFPIVDNS
tr|A0A1R1MKQ3|A0A1R1MKQ3_9BACT   47  WSTAFETFRAIKNFKHF.KQPELFKK...LFKLSEQEVSNFFS...KEVVKVKFPVVK.D
tr|A0A238Y624|A0A238Y624_9BACT   45  IRIQTALFFAIRQFAHL.KQLEIISE...ILNVSISSAEAFVN...NQIVQAYFPIVSED
tr|A0A285NI51|A0A285NI51_9AQUI   42  .DLKFVLYKTLRKYPYL.IDIPVLSK...TIKLPEKVIFKIFN...LPYKKAYFPVST.G
tr|A0A432RBX1|A0A432RBX1_9AQUI   45  .ELLFAIYSYFNKNPQH.LDLKKFSQ...ITKIPVFVLHKIFT...EKPIKVKFPVVN.N
tr|A0A2N7Q1Y2|A0A2N7Q1Y2_9AQUI    1  ........................................................IS.L
```

```
tr|A0A4R1GDP6|A0A4R1GDP6_9BACT    91   ...EGV....LIKALAFLSD..KTFS..........PK....EELSQVKEV......TQK
tr|A0A1E3G168|A0A1E3G168_9BACT    95   .NKSGK....IVEGLAIESS..VSFT..........NIPNAEEGVKIIENI......LGK
tr|A0A7C5U2L3|A0A7C5U2L3_9BACT    95   .NGSGK....IVEGFVFESD..KSYT..........NVAKAMEGIKILESI......LER
tr|A0A285P190|A0A285P190_9AQUI    91   ..GYSQ....MVQGMVIKDTS.QRIC..........NVEGIKRHIKSVEDLLKSRGLLKD
tr|A0A6C1BRW5|A0A6C1BRW5_9AQUI    90   ..D.GK....LIRGLAIKDTP.EVFC..........NLPEKKRYITPVVEYLRSKGFVSG
tr|A0A3M1C218|A0A3M1C218_9BACT    12   ..G.GR....IVKALAVPNTS.ENYS..........NIREQKRHIKPVTDYLRSRGVLSE
tr|A0A5P1V5W6|A0A5P1V5W6_9BACT    91   .VEKAKISVKPYKGLAIPETS.KIIT..........NLPELRSPLLTIKKF......LGT
tr|A0A1M6Q5V1|A0A1M6Q5V1_9AQUI    93   .GGPGK....IVRGIAVKGLS.KVVT..........NVGK..EKHQRLKKI......VGS
tr|A0A7V3ZWQ1|A0A7V3ZWQ1_UNCW3    89   ..GKGI....LSEMLVIKETS.EVIT..........NLRGIEESLKTVSNF......TKA
tr|D3SNA5|D3SNA5_THEAH            90   ..GEAH....LVKGLSVKGAGQSIIT..........NQKHLLKYMKELKEF......LKE
tr|A8UTR1|A8UTR1_9AQUI            89   ...GER....LVRGITIRGLE.SVIT..........NHEHLKGSMEVIKDF......LGS
tr|W0DEP5|W0DEP5_9AQUI            90   ..GKGE....LIKAVAIKNLK.EVIT..........NLKNIKRHFNPIKEF......LKT
tr|A0A7X7TR99|A0A7X7TR99_9THEM    95   .KALSK....IAHALVFEDD..AGYT..........NLAHQQASIDAIRKL......KGK
tr|A0A841GNB0|A0A841GNB0_9BACT    95   .G.NGK....IARAIVFESK..KSFT..........NNSDLKSSIEKIESL......LGK
tr|K2NM55|K2NM55_THEA7             1   ........................................MKSSLEKIESL......VGK
tr|A0A7V4KD75|A0A7V4KD75_FERPE   100   ...KVT....LARALVFKVD..SSFS..........NNPQVNDILAVLEKK......INA
tr|A7HMU9|A7HMU9_FERNB            97   .K.KSM....ITSALIFLSP..ISFT..........NIQGNRPNIEIIEKF......LGK
tr|A0A101EV44|A0A101EV44_9THEM    90   ...SAH....IRRALVFNSN..VEFC..........NLPELLEKLRFVEKI......VGF
tr|A0A432Q0P4|A0A432Q0P4_9AQUI    94   ...KGF....INTLLLVPLSSNEVVS..........TEPVDKANLELLSEL......IGK
tr|A0A1V5WQ44|A0A1V5WQ44_9BACT   100   ..SNGI....LAEVLIFPFSSTEFRAIC........FNKNYLEPATILAQL......TGK
tr|B0VHB4|B0VHB4_CLOAI           101   ..KNAF....LAKVFVYKLKSEEPIALA........ILGDKIENFSYLAQL......GKQ
tr|A0A238XV58|A0A238XV58_9BACT    86   NKQNDC....VLRGKIVY......TSKPPKRNSFLVDNNVIRACKTFKSLF......NTK
tr|C1DV83|C1DV83_SULAA           101   .EEEAH....LYKALIVEGTSKT..........FTFNKFVD...LQTIKAV......ANK
tr|B2V8L9|B2V8L9_SULSY            99   NPEESR....LNKALIIPLSNQT..........FTLNTFVNSQDLETIKEA......TNK
tr|C4FJQ9|C4FJQ9_9AQUI                 ............................................................
tr|F8E961|F8E961_FLESM           100   NGNESE....IQELIFFDVELESSTLTFPNENNH......VKDALISVSKA......LKR
tr|E4TFD9|E4TFD9_CALNY           100   ...QAE....LANLYVIELEDNCQTLSFPNTDMF......RLDYLQNIKKA......LGK
tr|A0A1R1MKQ3|A0A1R1MKQ3_9BACT    99   ..VKGE....IITAYLVPLKSS...........ILNFSKIDDELLSIIRKA......TGK
tr|A0A238Y624|A0A238Y624_9BACT    98   ..GNGD....IVTAFVVPLPSG...........LINFSHIPDEKLLPIVKS......TGK
tr|A0A285NI51|A0A285NI51_9AQUI    93   ..KI.......LELIALPL.EEIS.........VVLSSGVKEEDTRVIEKL......TGN
tr|A0A432RBX1|A0A432RBX1_9AQUI    96   ..KEAD....IASAYIFIFNKEIPQN......TFFNKEELKIIKRFLKQRN......INR
tr|A0A2N7Q1Y2|A0A2N7Q1Y2_9AQUI     4   ..QDGR....LCKAIVFIFDDDINVTTLPHTNSLNHKEGIEAVRKIFKKRY......PNK
```

```
tr|A0A4R1GDP6|A0A4R1GDP6_9BACT   122   EFGFFYN.R.EFSGESFQAPLAYALL....YGELPKRVILSGKLLPKGNFKA..ENAKEK
tr|A0A1E3G168|A0A1E3G168_9BACT   132   KLIVLFS.E.PFSGNSFMLPLYLSVATCGKIQEMLPHILFTGGFKSLHGLLP.TDYVDAK
tr|A0A7C5U2L3|A0A7C5U2L3_9BACT   132   KLIVIFS.D.DFSGNSFMLSLYIAIRTGGKIRKLFPKLIFTGAFTKALVPEP.TDHVDVK
tr|A0A285P190|A0A285P190_9AQUI   134   YLSVFLN.S.YITGKSFQLSLALSLL....TEKVPDRFCFTGGVDAKGNVQA.VDNIPQK
tr|A0A6C1BRW5|A0A6C1BRW5_9AQUI   132   SVSVIFD.S.EFVGNSFQLSLTLALCMDAEKKRLPPNLCWSGGVRKDGSIVK.VDSLDKK
tr|A0A3M1C218|A0A3M1C218_9BACT    54   GLSVFFD.S.GFVGNSFQLAMVLSFL....VKKLPSDLCWTGGVRKDGRLAK.VDSLEKK
tr|A0A5P1V5W6|A0A5P1V5W6_9BACT   133   SFAVFFE.T.YFTGKSFMLPLAVALS....IAKIPEDLRFTGALNSKGDLLE.VDHIKEK
tr|A0A1M6Q5V1|A0A1M6Q5V1_9AQUI   129   GFAVFYD.T.EFTGKSFQLPLAVSLM....SERIPEGVLFTGELSQDGRILR.ANHIEEK
tr|A0A7V3ZWQ1|A0A7V3ZWQ1_UNCW3   126   KFAVFFK.D.EFVGESFMLPLAISLV....TKNIPDDIVFTGKIDEEGNVYD.VGKIMEK
tr|D3SNA5|D3SNA5_THEAH           128   GFGLFFE.E.DIKGESFLLPAAVSLY....IENPPEDAVFTGRVDREGKIYT.VDNISKK
tr|A8UTR1|A8UTR1_9AQUI           125   GFGLFFD.E.EFSGESYMLPAVVSLY....IENPPPKVVFTGKIDKEGNIYE.VNGIPKK
tr|W0DEP5|W0DEP5_9AQUI           127   GFAVFFD.R.EFAGASFQLPTVLNLY....VENLPQDALFIGAIDKKGNIKS.VDGIEEK
tr|A0A7X7TR99|A0A7X7TR99_9THEM   132   NFSVVFD.T.LFYDDSFMFAVAVGAL....SKNVPENIAFTGRIGEHGNILP.INSLSEK
tr|A0A841GNB0|A0A841GNB0_9BACT   131   KLIVIFD.S.EFKGNSFELAVAIGSL....CKKIPKNIAFTGEIDEKGNIKRNIEYLDLK
tr|K2NM55|K2NM55_THEA7            15   KLIVIFD.S.EFKGNSFELAVAIGSL....CKKIPKNIAFTGEIDEKGNIKRNIEYLDLK
tr|A0A7V4KD75|A0A7V4KD75_FERPE   135   KISVLFD.R.DFVGESFELAVAVGRL....VEHIPENLAFTGRVEEDGKILK.VENFYEK
tr|A7HMU9|A7HMU9_FERNB           133   NLAVVFP.E.EIVGDSYMLSITIGSL....TKRVPDHIVFTGGILEDGTITS.ADNILEK
tr|A0A101EV44|A0A101EV44_9THEM   125   ELSVLFD.G.DLCGDSFMLAVAVGAL....SKNLPGHVAFSGSLDKSGNVGL.VENLEKK
tr|A0A432Q0P4|A0A432Q0P4_9AQUI   131   GFFVTFEKNFNFKTNSYTLALYAGLV..YGKE..AGKFAFTGKVTTGGKLEE.VENVTQK
tr|A0A1V5WQ44|A0A1V5WQ44_9BACT   140   SFLMVFT.E.DFVGDSWMAATSAALI..ADNPDDLKDFCFSGAVNESGKILS.PAQLTEK
tr|B0VHB4|B0VHB4_CLOAI           141   NCLIGFN.K.NIQGNSWQLAVLATLL..VKDEKIISKIAYSGIVLPSGEIIT.AEEIEYK
tr|A0A238XV58|A0A238XV58_9BACT   130   NFILVFDGR.EFTGTSFMLSVVVAHF..FSQK.LFEYFMFTGEVLPSGKINK.VDSILEK
tr|C1DV83|C1DV83_SULAA           137   DFFVIFS.N.YFTGDSYQFSIVAGLI..AKDKNILKNLAFTGKVSSSGKILP.VNHVNEK
tr|B2V8L9|B2V8L9_SULSY           139   NFFVIFD.N.IFSGKSYQLAVAAGLI..AKEKEILDNVAFTGEVSSNGFIIP.VNHLEEK
tr|C4FJQ9|C4FJQ9_9AQUI             1   ...MIFD.N.IFSGKSYQLAVTAGLI..AKEKEILDNVAFTGGVSSNGFIIP.VNHIEEK
tr|F8E961|F8E961_FLESM           144   NFFIMFD.N.YFAGRSFSLAAAAAGL..LKED.KLKYFAFSGEVKENANIAK.VENLPAK
tr|E4TFD9|E4TFD9_CALNY           141   NFFVFFD.R.FFTGRSFGLALAACFF..IKDE.IREKLLFTGEVRADGRVYD.VDGIGLK
tr|A0A1R1MKQ3|A0A1R1MKQ3_9BACT   136   GFAIAFD.S.PFSQDSFMLAVAAGLL..TEDKCFLSHLSFTGSVDYYGNILS.VGFIGEK
tr|A0A238Y624|A0A238Y624_9BACT   135   GIAVAFT.H.DFCQESFMLSVCAALI..SEID..ITYLAFTGRVDSFGNVLT.VNNIGQK
tr|A0A285NI51|A0A285NI51_9AQUI   128   KFFSIFS.D.RFEGSSYMLALYSSLK..YSQE.ILKSFSFTGVVGADGEIYE.VGFLPEK
tr|A0A432RBX1|A0A432RBX1_9AQUI   138   NFFVIFD.K.QFKGKSYLLAVVAGLI..LPEY.ILEDFAFTGEVNEEGEIYP.VGYIKNK
tr|A0A2N7Q1Y2|A0A2N7Q1Y2_9AQUI    52   DFFVMFD.S.NFKGSSFTLALLSALF..LKEE.TLKKFAFTGDVKESEDILK.VNYLDQK
```

```
                                                   .              .           .             .              .
tr|A0A4R1GDP6|A0A4R1GDP6_9BACT  174  EEVATKEGKFLIAE..G.....NIHEIKEFFSKE.EKDIPLLIATGKRE..ENIPNFEIF
tr|A0A1E3G168|A0A1E3G168_9BACT  189  QKTANAEGKELVII..DELG..DLRKFSNFITSE.EKHVPLFVGSKITQ.KKAEDEYKKL
tr|A0A7C5U2L3|A0A7C5U2L3_9BACT  189  HEISKKLGRRLVTI..EEID..DLNNLVMFFMKD.KKDISFYFSVRSDR.DSALSEFRNF
tr|A0A285P190|A0A285P190_9AQUI  187  EKACRSSGKKLISP..VNVR..SVDDIIDWFSKP.FVDVPFVITKERS.....RPNIGDF
tr|A0A6C1BRW5|A0A6C1BRW5_9AQUI  189  SEVCERFNMHLAMP..FHLP..KVDDLLNWLSAN.IVEVPVAVSIDHL.......RLEEF
tr|A0A3M1C218|A0A3M1C218_9BACT  107  AQLCQMEGKRLAMP..FHLR..SVDELTDWLNAE.LVDIPLAVSKDPI.......KLEEF
tr|A0A5P1V5W6|A0A5P1V5W6_9BACT  186  LNYTKQNNLRLITP..LQVK..HFNAIKTYLEND.RWDIPFYVTSSGKE..EFNMFLNSY
tr|A0A1M6Q5V1|A0A1M6Q5V1_9AQUI  182  KKIAAREGYILVDP..AKLPKPDLKSLLNWLNKD.IHHIPMLITSS..E..KHQDELKDF
tr|A0A7V3ZWQ1|A0A7V3ZWQ1_UNCW3  179  QKACENAGKRLLYS..LYIP..TVNFAKEWLDRE.SFDIPFYVTTTT.Q..FPEKEFEAF
tr|D3SNA5|D3SNA5_THEAH          181  RKAAQKEGKRLIDS..SGIK..SLQELKEWCDAK.EHHVPFMVTTKGSE..DWLSKWRDF
tr|A8UTR1|A8UTR1_9AQUI          178  RALAQKEGLRLIEP..SYLD..SVRALKDWLDAE.RYHIPFYIT.KSKE..NYEGELQSF
tr|W0DEP5|W0DEP5_9AQUI          180  KKLAKELGLRLVEP..YYLS..TVDDLKAWFDAE.SYDVPLYIT.KTQD..RWEGEFKSF
tr|A0A7X7TR99|A0A7X7TR99_9THEM  185  EEVVKDENLILLSP..LDAA..HIDDVIDVLNAQ.GASVPVFYTYQDND..DADRKYESF
tr|A0A841GNB0|A0A841GNB0_9BACT  185  LKICSENNLKLISA..LDVD..NVFELKEFFEAK.KIHIPILISLMAKDKEYIRTSYEEL
tr|K2NM55|K2NM55_THEA7           69  MKICSENNLKLISA..FDVD..NVFELKEFFEAK.KIHIPILISLMAKDKEYIRTSYEEL
tr|A0A7V4KD75|A0A7V4KD75_FERPE  188  VVYCNKNNMGLISY..IDVS..HISEIIDFLNER.EFHIPILLRFSSKP.QDIEILWKKL
tr|A7HMU9|A7HMU9_FERNB          186  IEVCRRNGYVLITS..DDAK..NVVDLQKFFEKK.KYHVPVYISFNSSK....NDYWDML
tr|A0A101EV44|A0A101EV44_9THEM  178  FQVCSEQGLELLTG..LDVQ..NFRELVDFFNTR.EHHVPVYLCYKHSE.RIEDRGWEEL
tr|A0A432Q0P4|A0A432Q0P4_9AQUI  186  LELCRNSGIPLIFAKRRDME..SVEDLEEFLTKL.QIPIFIFPSKEK....EREAFLTNF
tr|A0A1V5WQ44|A0A1V5WQ44_9BACT  195  KQCCEVYGKRLIT....AVK..SLEELEFWLNSC.ELPVPLVQSSGT.P.DKKSSWLLKM
tr|B0VHB4|B0VHB4_CLOAI          196  KRCCQNL.....VH....RIK..KIEQLDAWLNTE.TIPLPVIQYQGE.E.NELKRWQKAM
tr|A0A238XV58|A0A238XV58_9BACT  185  KKLAQRYGKKLISP..EDVS..SIEEVDFWLNESKPLPVPFSVMIKKNN.DG....LKLL
tr|C1DV83|C1DV83_SULAA          192  EKITKANEKNLITP..DDIS..TLEELEFWLNSS.QIPVILLNR..NTD.NNIKESLHQI
tr|B2V8L9|B2V8L9_SULSY          194  KEITEKAKKVLITP..EDIE..NLEELSFWLNPE.HLPVIFIHI..NKP.ELALQSLKQM
tr|C4FJQ9|C4FJQ9_9AQUI           53  KEITEKAKKVLITP..EDIE..NVEELNFWLNPE.HLPVIFIHI..NKP.ELALQSLKQM
tr|F8E961|F8E961_FLESM          198  RKISEEKDLFFVSP..DSVD..NLNQLTK.LNAE.TVDIPFIQLFGKQK.TELEKNLEKI
tr|E4TFD9|E4TFD9_CALNY          195  TEIAKANKRYLVGS..THVN..SITELKY.LDRN.CIDVPFIQLFGKSK.NELNKNFSKL
tr|A0A1R1MKQ3|A0A1R1MKQ3_9BACT  191  EKISEEQNLKLITA..EDVR..NLEQLKCYLKR..NIPVPFPLMIGKPK.ETVKIFLRSF
tr|A0A238Y624|A0A238Y624_9BACT  188  EVISRKSGKILITP..EDVN..HLSLLKKHLGK..SLDLPFPVVIGKPE.TAVDLFFKSF
tr|A0A285NI51|A0A285NI51_9AQUI  182  EKASKMAGLKLISP..LLVS..NVKELDYWLGEE.SIDIPFLYLRKKKH.PE..KVLENF
tr|A0A432RBX1|A0A432RBX1_9AQUI  192  KKIADKNNLNLITY..EDIY..HIDELIYYLGEE.AIDIPFVQLSNKTE.EEAYISLEKL
tr|A0A2N7Q1Y2|A0A2N7Q1Y2_9AQUI  106  KKIAQDAQLKLITY..EDVS..NVEELLYYLGEG.PIDIPFININRG.L.DEALNSLEKL


                                                   .                  .              .            .
tr|A0A4R1GDP6|A0A4R1GDP6_9BACT  224  CKNVGFK..P.........LKGLLDEEKLIV.ELPTFLPH......DRDWRELFLPI.
tr|A0A1E3G168|A0A1E3G168_9BACT  243  HTAVLET..K...NV.PTPELLVKLL.GEEPYVCWEGDL..D......VSQFEDIAQEI.
tr|A0A7C5U2L3|A0A7C5U2L3_9BACT  243  CNDVSSF..L...DLKFNGNMLDKVF.ERSTWLFWESEL..S......SQDFVIAADEI.
tr|A0A285P190|A0A285P190_9AQUI  237  WE.........EEHVLKNLKHFHEIT.EEDLIL.ETGQL..E......GQKWQEVCTEF.
tr|A0A6C1BRW5|A0A6C1BRW5_9AQUI  237  FH.........KEENLLNLKNIHRID.PSKLVI.QTGQL..S......GIRWQETAKRF.
tr|A0A3M1C218|A0A3M1C218_9BACT  155  FE.........KRENLLNLWHIHRID.PSELIL.HTGRL..E......GELWKETARRF.
tr|A0A5P1V5W6|A0A5P1V5W6_9BACT  239  TGTKVLA..E..FEILKGLELFYNLQ.EDNFYI.ITGQLN.S......KEDWEKTCKTF.
tr|A0A1M6Q5V1|A0A1M6Q5V1_9AQUI  235  TRNLQIE..D.LNLELAYLETFG.....IEPVL.WTGQI..K......DQRWQEVCKEF.
tr|A0A7V3ZWQ1|A0A7V3ZWQ1_UNCW3  231  CEAARID..L...QELTKGLTFYSIN.LEDLFM.VTGSLS.E......EEDWITNVERF.
tr|D3SNA5|D3SNA5_THEAH          234  LSYMKD.....SENIVRKLEIINGIT.AEDLVL.YTEQLPK........GDWTKYMMDF.
tr|A8UTR1|A8UTR1_9AQUI          230  HSHLTFE..E.PEKTLDLLEVINGIP.KERLVV.LTGQIPPE......ESVWEEIVREF.
tr|W0DEP5|W0DEP5_9AQUI          232  LKATGIS..K...EQLTKLEVLSGLE.TKP..I.ITGQL..A......GDVWKNVLEEF.
tr|A0A7X7TR99|A0A7X7TR99_9THEM  238  VEFVHSV..QDNCVGISGIRLAEKVF.GFSTLL.KYKKL..E......FTDWNELAIVG.
tr|A0A841GNB0|A0A841GNB0_9BACT  240  KEAVNEK..F....EIKYGDIFEKIY.DVNKVFVK..DS..L......NDDWGQVLKEV.
tr|K2NM55|K2NM55_THEA7          124  KEAVNEK..F....EIKYSDIFEKIY.DVNKVFVK..DS..L......NDDWGQVLKEV.
tr|A0A7V4KD75|A0A7V4KD75_FERPE  242  KERVLAF..SG.KGGASNCNLFERIY.KAQLIYSISREL..S......EDEFYEHIEKA.
tr|A7HMU9|A7HMU9_FERNB          237  FEEVKEK..F....YVDDIKLFKKVY.NPEFKY.ISSEL..R......VEDFQEEIGKV.
tr|A0A101EV44|A0A101EV44_9THEM  232  KEVVEKS..F....PTGSLELYRRIY.RVDMFH.KRYML..K......KEEFAQELKRA.
tr|A0A432Q0P4|A0A432Q0P4_9AQUI  239  K............FSQNYIKSVFHLE.EELSYIKPFNETPED...FEEYIQWLKRIGE..
tr|A0A1V5WQ44|A0A1V5WQ44_9BACT  246  EAAIQKE..Y.PLFNLQALFEFTGLN.VECLSIFKEGDFPFI......PEVWQAFLGG..
tr|B0VHB4|B0VHB4_CLOAI          243  EQKVQEK..F.SWFSYELLEDFYGIT.NSDLAIFGNGILPFE......ANAWQKLLQEQV
tr|A0A238XV58|A0A238XV58_9BACT  236  ESAIKER..E.PLFSLKNLEVFFEIK.REDISILHSGFLSEN......YQEWLAFVHRI.
tr|C1DV83|C1DV83_SULAA          244  ETLIKQD..C.PYFTIKNLIKFYNLS.EEDLYIITPSIDFSN......REELLNILKQF.
tr|B2V8L9|B2V8L9_SULSY          246  EDAIKKDERF.KYFKLENLKKFYRLE.DQDMYLITPSVDFSN......REELIKILNEF.
tr|C4FJQ9|C4FJQ9_9AQUI          105  EDAIKKDERF.KYFKLENLRKFYRLE.DQDMYLITPSVDFSN......REELIRILNEF.
tr|F8E961|F8E961_FLESM          251  SGNEI.......VNDYKIWVGILGGD.KSLVFTHTEEMLENT......TEVWDELLLDF.
tr|E4TFD9|E4TFD9_CALNY          248  K..EF.......ISDGRVMVDVLGFS.DENLSVFCEDFIENN......IDTYLNYLRDF.
tr|A0A1R1MKQ3|A0A1R1MKQ3_9BACT  244  EKETK........LNCDAVSKFSNIS.LSDMGIVKRNYLENS...LETFHKFL..EEEF.
tr|A0A238Y624|A0A238Y624_9BACT  241  SEKTG........IQPDLLAKIYKID.KAVMGIHMSERIENS...PEVFHKII..DTAK.
tr|A0A285NI51|A0A285NI51_9AQUI  234  EKIIKEK..R.ENFSLRGLESIFDIH.EEDLFINLQKDLPS.VKNSETLWQWKNEIKNF.
tr|A0A432RBX1|A0A432RBX1_9AQUI  246  ENKIKEK..I.NFYSLEKLEKFFGIS.KKDLILTSEYLPKPE....KDNNQWINTIKTF.
tr|A0A2N7Q1Y2|A0A2N7Q1Y2_9AQUI  159  ENAMKEQ..I.KFFSLEKLSKFFNID.KEDLILTTESLPPITEEDLSKENPWIKAVLEF.
```

```
tr|A0A4R1GDP6|A0A4R1GDP6_9BACT   263  REKVLKLKE...FL.....PDLSLHVALKAPITFSLGAGAVIGTGKVP.VAVYHFEN...
tr|A0A1E3G168|A0A1E3G168_9BACT   287  VEFLRGDRFKIQKV.....GDFIPHIAMKVPISLAFLVGLKLKPY.RK.LVLYHFDT...
tr|A0A7C5U2L3|A0A7C5U2L3_9BACT   288  IDLLTEETFGK.........DTVLHIAIKGPSALAFIVGLKLKPY.RE.LVFYHYNS...
tr|A0A285P190|A0A285P190_9AQUI   277  INRIRRMDYE..LS.....NRLRLNLVINGPTALAMALGILYSHT.RP.FRVFHYSN..S
tr|A0A6C1BRW5|A0A6C1BRW5_9AQUI   277  FGLISVLDYT..LI.....GRLKAHIVVNGPASLSFALGILYGHT.RP.SVFYHYHS..S
tr|A0A3M1C218|A0A3M1C218_9BACT   195  SQLVKRLDYT..LE.....RRLRAHLVINGPVSLAFALGVLYGHT.RP.SAVYHYLR..G
tr|A0A5P1V5W6|A0A5P1V5W6_9BACT   285  AEKIDRIKTC..LP.....GIKTYHLGLRGPVSLGFAFGVLFSHF.DP.FVFYHYQTIEG
tr|A0A1M6Q5V1|A0A1M6Q5V1_9AQUI   277  YSRLKHIGSV..LG.....G.FHLHIAIRGPAALAFALGILHGSF.KP.FTIYHFQN...
tr|A0A7V3ZWQ1|A0A7V3ZWQ1_UNCW3   276  YRRISEISHK..LS.....SRECIHFGMRGPATLAMAMGMVYGCQ.KP.LTLYHFQN...
tr|D3SNA5|D3SNA5_THEAH           278  YRKLTDVEKR..LK.....GKVIFHLAIDGPASFAFGLGILFGSQ.KP.FTVYHYQN...
tr|A8UTR1|A8UTR1_9AQUI           278  YLRVKEIESA..LG.....AREVLHIAMNAPSALAFALGAVFGSQ.KP.FVFYHYQN...
tr|W0DEP5|W0DEP5_9AQUI           274  WRRFKETEQK..LH.....NKERFHIAINGPVALAFAIGVLFGSQ.KP.FVFYHYQN...
tr|A0A7X7TR99|A0A7X7TR99_9THEM   285  GDNLRMI.....AK.....AGFIPNIAFEGPGPLAMGVGIRFGAQ.YP.IVIYHKVA...
tr|A0A841GNB0|A0A841GNB0_9BACT   282  NSLLYKI.....IS.....NRGIPHIAIMGPASFSMALGIALGVQ.NP.LVVYHKAG...
tr|K2NM55|K2NM55_THEA7           166  NSLLYKI.....IS.....NRGIPHIAIMGPASFSMALGIALGVQ.NP.LVVYHKAG...
tr|A0A7V4KD75|A0A7V4KD75_FERPE   289  YGIIRNV.....ID.....NSGIPHIAINGPAAFALGLGITLGAK.DK.LVVYHYQG...
tr|A7HMU9|A7HMU9_FERNB           280  KNILDRI.....IS.....SSGIPHISLNGPATFAMGLGMAIGST.HL.LAVYHFQG...
tr|A0A101EV44|A0A101EV44_9THEM   275  FDTLQRL.....FQ.....SGGVPHLAINGPSSFAMGLGVAIGVK.RK.MAVYHYQG...
tr|A0A432Q0P4|A0A432Q0P4_9AQUI   281  ..EIKTLKER..YL.....PSITVGIT.SNPVVFSFIAGVIFSKN.RLNTVFYKYLK..E
tr|A0A1V5WQ44|A0A1V5WQ44_9BACT   294  IDHFDRVEQK..LGT....RKAIFWYA.GMLSSLQFGIGAIFGFN.RP.IYICHWEV..S
tr|B0VHB4|B0VHB4_CLOAI           293  KDKFKLLEDK..VMP....KKVLWFYA.GQISTLQLGIGALFGFK.RA.VSILQMEF..S
tr|A0A238XV58|A0A238XV58_9BACT   285  REQLRKLFSN..EKL.VELKKPVINLI.CSESIISFVIGVLVGTK.KR.VVFHSYN....
tr|C1DV83|C1DV83_SULAA           293  EERLEKLFSV..RNS.......ILYIS.LSVASLGFLVGSLIGAR.KK.VVILHYQ....
tr|B2V8L9|B2V8L9_SULSY           297  REKVSKLLTL..EGVIKDHNKVVLNIS.AGISTLALYFGVILGNR.QA.SIIYHYQ....
tr|C4FJQ9|C4FJQ9_9AQUI           156  REKVSKLLTL..EGVIKDHNKVVLNVS.AGISTLALYFGVILGNR.QA.SIIYHYQ....
tr|F8E961|F8E961_FLESM           296  YEKINKLYQL..P......YYVNIHFL.GSLSAFAFLSGIVFGAK.NK.ITIHHYQ....
tr|E4TFD9|E4TFD9_CALNY           291  YYKVKRVYEI..D......FEVNLHLL.GSLSSFAFLMGLVLGAK.KR.FVIYHYQ....
tr|A0A1R1MKQ3|A0A1R1MKQ3_9BACT   289  SSKLLKMTRQ..R......EEAVFHII.GVISSLMYGAGIIFGVR.KK.AVLYHYQ....
tr|A0A238Y624|A0A238Y624_9BACT   286  .ENLAEIYRK..I......PQATVHIT.GTISTVMFGIGVAFGIR.RR.FILHHFQ....
tr|A0A285NI51|A0A285NI51_9AQUI   288  ERKITQIYSR..IKG....KKRILHLA.SSIASLSLGFGVKLGGR.KP.VILYHYQ....
tr|A0A432RBX1|A0A432RBX1_9AQUI   297  EEKLKKIYSS..IKR....KKRILHFS.GSISSLAFGFGIKLGTK.KP.VIVYHYQ....
tr|A0A2N7Q1Y2|A0A2N7Q1Y2_9AQUI   214  ESKLKNIYDK..VGS....RKRVIHFV.GSISSLVFGLGVKFGSR.KP.VVVYHYQ....
```

```
tr|A0A4R1GDP6|A0A4R1GDP6_9BACT   311  .GSYHRVIDLRK..DSRRIKRR...KR....KLS.FIEV...EE.....EVRGSDTAVIA
tr|A0A1E3G168|A0A1E3G168_9BACT   337  .DKYFPVLDLSS..NPREVASR...NVSQG.NFK.KIEFSLKNLTNDSNTVLSNHEFAII
tr|A0A7C5U2L3|A0A7C5U2L3_9BACT   334  .GKYSPVLDLRE..NPRMIVER...IR.YE.SFE.KIQV...ETYDDSLICCQDSEVAVL
tr|A0A285P190|A0A285P190_9AQUI   326  EKRYYTI....DVFNTRELKER...VK....EYT.HTEA...EL.....DSSEGKDLAVI
tr|A0A6C1BRW5|A0A6C1BRW5_9AQUI   326  ERKYFPI....DLQNTREIKEH...TR....DYQ.FVKS...EL.....KE.GGEDLAVV
tr|A0A3M1C218|A0A3M1C218_9BACT   244  DKRYYAV....ELFNTREVKEA...VG....EYR.HIRQ...DL.....EG.DGEELAVV
tr|A0A5P1V5W6|A0A5P1V5W6_9BACT   336  ETKYHPI....KVEEPRFFKER...LK....VYQ.YLKP...SF.....E.KKGEDLVIV
tr|A0A1M6Q5V1|A0A1M6Q5V1_9AQUI   324  .NAYRPI....VVDNVRYIKER...LS....GNPRLINY...TV.....T.GSGEEVAVV
tr|A0A7V3ZWQ1|A0A7V3ZWQ1_UNCW3   324  .GKYVEI....PVPNVRYLKDR...GH....SYN.MVKF...SF.....EPGKIEEVAVI
tr|D3SNA5|D3SNA5_THEAH           326  .GKYYPI....EVENVRELKQR...ME....LSEITLKW...SL.....E.SRDDRLAVV
tr|A8UTR1|A8UTR1_9AQUI           326  .GDYKPI....KVENVRSLKER...LT....DYK.HIDH...QL.....E.GEGRELVVA
tr|W0DEP5|W0DEP5_9AQUI           322  .NIYHPI....TVENVRELKER...KE....SLE.KIEQ...HF.....Q.KGGKSLVVM
tr|A0A7X7TR99|A0A7X7TR99_9THEM   330  .QGYAKVIDLSD..NLRKIKAI...KQ....SFD.RFDV...EV.....S.GDGDICVYI
tr|A0A841GNB0|A0A841GNB0_9BACT   327  .IEYKPVIDLTD..NVRKVKNI...V...S.DYK.FVKYS..VE.....N.ENGKNCAFI
tr|K2NM55|K2NM55_THEA7           211  .IEYKPVIDLTD..NVRKVKNI...V...N.DYK.FVKYS..VE.....N.ENGKNCAFI
tr|A0A7V4KD75|A0A7V4KD75_FERPE   334  ..NYIPVLDLSTEQNLRLIKTV...TRFKE.ALQ.ELTCEVLEH.....N.TNKRKAILA
tr|A7HMU9|A7HMU9_FERNB           325  ..EYHLVLDLTEPENLRKIKEQ...K..N..NIR.FLNY...RI.....E.GTGKRAAFI
tr|A0A101EV44|A0A101EV44_9THEM   320  ..GYHLVLDLTKPENLRRIKAL...KKEE..ELE.LLNY...EL.....I.DDGEEAAFV
tr|A0A432Q0P4|A0A432Q0P4_9AQUI   328  GKVYKSLFTIESD...RDIPET...EGARN.....LIK..IFK..........NEG...EM
tr|A0A1V5WQ44|A0A1V5WQ44_9BACT   343  NQEYLPVFRLYRPNNARVLKNVSFDPHSFT.....HIKYEIDA......ISNQMNDLALI
tr|B0VHB4|B0VHB4_CLOAI           342  NTTYHEVFILYGKENARQLKNVSVKKEDYQ.....YIQSELLI......NEPHKNELGFI
tr|A0A238XV58|A0A238XV58_9BACT   335  RGDNVYVPSMETDDSLRSLDFP...GAEFCGE...LIDYEYFI......SDKNKNVLPVI
tr|C1DV83|C1DV83_SULAA           337  .GEYRKAIDMSKD..PRVIKEN...VKEYS.....II...QPE......SCNTDQEIALI
tr|B2V8L9|B2V8L9_SULSY           348  .KEYHKVIDLTDN..PRKIKEK...KSEFE.....KI...SVN......K.NIQDPLMII
tr|C4FJQ9|C4FJQ9_9AQUI           207  .KEYHKVIDLTDN..PRKIKEK...KSEFE.....KI...SVN......K.NIQDPLMVI
tr|F8E961|F8E961_FLESM           341  DGSIFRVMDFSEK.SVRLLKSK...TKKYE.....KVKYSVEY......TVAESEDAAIV
tr|E4TFD9|E4TFD9_CALNY           336  DGGVHKVFDFMKQ.SVRVLKSK...KNHYE.....YLKFQIFY......NDPDSDELLVS
tr|A0A1R1MKQ3|A0A1R1MKQ3_9BACT   334  DSKYYPIITINRE.....LKEI...KRSRN.....WIQTKQIE......KG.SNKETAIT
tr|A0A238Y624|A0A238Y624_9BACT   330  DGQYIPVIETSRS.....LKEI...KEKLT.....YVKTKKLK......DG.TENELVLI
tr|A0A285NI51|A0A285NI51_9AQUI   335  SDEYIPVIDLSDSKKIRKIKYV...RKNIDTELK.YITVSYPA......SIEKSQDVAVG
tr|A0A432RBX1|A0A432RBX1_9AQUI   344  ADNYHKVIDLSNEEQIRQIKHI...KEKLE.....NLEIKQLK......GNKNAKELAIA
tr|A0A2N7Q1Y2|A0A2N7Q1Y2_9AQUI   261  ADTYNMVLDLSDEDKLRRIKHI...KENLE.....LLKLENIK......SA.NQEEVGVV
```

```
tr|A0A4R1GDP6|A0A4R1GDP6_9BACT  352  LQVASHETRS.KGEELSQKF...NADFFYVNA..PELKGSLPLDL...........DWAE
tr|A0A1E3G168|A0A1E3G168_9BACT  389  IDMAGRSIQD.AVEKFLLTN.DKKMQVVTIT...HKNSGCVPLS............NWSE
tr|A0A7C5U2L3|A0A7C5U2L3_9BACT  382  IDMAGQNAID.EVRMFLREN.GICAKLLHIT...HKDSGNIPVG............DWSK
tr|A0A285P190|A0A285P190_9AQUI  366  IRGAHHDPTG.DVKAYLTKE.GISADILVLSP..KEKEGNIPPN............QLKE
tr|A0A6C1BRW5|A0A6C1BRW5_9AQUI  365  LFFSHHNPTA.DVEHFLESK.GIKADLLLLTT..ESYRGNLEPS............TFKR
tr|A0A3M1C218|A0A3M1C218_9BACT  283  LYFGHHNPVA.DIKAFLADR.GIGAEVLLLWM..EEGRGNIDPA............DFKP
tr|A0A5P1V5W6|A0A5P1V5W6_9BACT  375  LNFSHHEPTA.DVKKYVSSI.LR.EPSFLILE..TEFKGNLPIE............TFRE
tr|A0A1M6Q5V1|A0A1M6Q5V1_9AQUI  363  INLAHHSVQL.NSLP.......LTNATYLVIE..TQGGGNLPVE............NFKD
tr|A0A7V3ZWQ1|A0A7V3ZWQ1_UNCW3  363  LSFAHHEALA.DAKNYIVKN.LKDKPAFLVIE..HERKGNLEPL............EFVD
tr|D3SNA5|D3SNA5_THEAH          365  IDLAHHTSIG.TVKAYIDD....GMSLLHVE..HPHKGNLKVE............EISQ
tr|A8UTR1|A8UTR1_9AQUI          364  FAIAYHDPVA.SVKEFTQG...M.DSTLLVVR..HKSSGNVPVE............EMFE
tr|W0DEP5|W0DEP5_9AQUI          360  LSFAHHEMES.DVKNYISRK.VE.NPSYLLLR..AKSSGNIAVE............DMKE
tr|A0A7X7TR99|A0A7X7TR99_9THEM  370  IKTASHELKA.QVIDFVRKK.EGNNFMYIYAS..HKNSGNLPVE............DWTV
tr|A0A841GNB0|A0A841GNB0_9BACT  368  LYFASHNPYD.AVKNFLSKN.NIDSKLVLIEP..IFGKGNLPAE............DWSE
tr|K2NM55|K2NM55_THEA7          252  LYFASHNPYD.AVKNFLSKN.NIDSKLVLIEP..NFGKGNLPAE............DWSE
tr|A0A7V4KD75|A0A7V4KD75_FERPE  381  IDLASHTLLS.SVREYAKEN.IPDGFIIHVMPKNMEASGNIPLG............DWKK
tr|A7HMU9|A7HMU9_FERNB          366  IQIGSHDPYP.DAKSYIQRH.LPDTAIVHIRA...NSSGNLPLG............NWTE
tr|A0A101EV44|A0A101EV44_9THEM  363  IHLASHDPLP.QVKEFLKG...KDVLIVYVRS...KQPGVLRIG............DWTE
tr|A0A432Q0P4|A0A432Q0P4_9AQUI  363  ITVTFNNRVDTDGIVF............KIP......SGELLDKH.........SKALAY
tr|A0A1V5WQ44|A0A1V5WQ44_9BACT  392  LYFGSHNPYQ.EVINYFKPL.FPDSGTLFISL..KESQGQINLDS...........DWLR
tr|B0VHB4|B0VHB4_CLOAI          391  IYLGSHNPIG.EAKAYCQKQ.LQINNFLIIQA..RENQGVMETSQ...........NWLP
tr|A0A238XV58|A0A238XV58_9BACT  383  IDMAPTTRIEYDVKEFLKKNRIENFIVIKIDR..DKIKNYIPED..........TNIRKK
tr|C1DV83|C1DV83_SULAA          377  LNIASHNPVN.SAKSYIEKN.LPHVKSTCVIN..TIYGGNIPLE............EFLT
tr|B2V8L9|B2V8L9_SULSY          387  IYLASHNPIE.KGLELKEKL..RAKGELIIQS..KEHQGNLEIG............DWSD
tr|C4FJQ9|C4FJQ9_9AQUI          246  IYLASHNPIE.KGLELKEKL..GAKGELIIQS..KEHQGNLEIG............DWSS
tr|F8E961|F8E961_FLESM          386  IYLASHNPKN.DAQKYIESH.LKC.SLLFICL..ENNQGNIDLN..........EDDWIK
tr|E4TFD9|E4TFD9_CALNY          381  LYIASHNPVN.DAAKFAKEK.LNC.NMVEIRL..KEQQGYLPLDN..........EKIWID
tr|A0A1R1MKQ3|A0A1R1MKQ3_9BACT  374  IHIASHNPLG.STRIFVENY.LKNSSHFYITL..KEKTGNVPLN..........QEIWKT
tr|A0A238Y624|A0A238Y624_9BACT  370  LHIASHNPVS.TVMDFSDKK.LKNMPVYTITL..KDSLGNLPID..........GKLWTE
tr|A0A285NI51|A0A285NI51_9AQUI  385  IWLASHNPFD.SIERFLKTN.NLTDCLVKIES..KNFQGDIPLPSDFED...TPRNYWIE
tr|A0A432RBX1|A0A432RBX1_9AQUI  390  IWIAGHNPYG.DVLRFSENK.NWE..VIGIEA..KKFKGNLPLPKKREY.....ENLWLE
tr|A0A2N7Q1Y2|A0A2N7Q1Y2_9AQUI  306  IYFASHSPLG.DVLRFSENK.NLD..IFLIEA..KDLKGNIPIPKKNEENFFTLLNLWSE
```

```
tr|A0A4R1GDP6|A0A4R1GDP6_9BACT  395  VVA.EIYEAFNR..I.YAKGHKKFHLVMSVPNPIAFALGMAVGN.YWDVTVWSYFK....
tr|A0A1E3G168|A0A1E3G168_9BACT  432  EVA.EIKSVINM..FNSSKT..VYHLFFSCPVPIGFGLGLALKEDTHKLNVYHFFQ....
tr|A0A7C5U2L3|A0A7C5U2L3_9BACT  425  EVR.EIKTLLDR..V.GKKV..IYHIFLSCPVPLAFGLGLSMKEDTHKVKLYSYSR....
tr|A0A285P190|A0A285P190_9AQUI  410  VAK.EYASHIQS..ARAQKHYENIHFFLSTPVAIGYLLGVAFGH.YTKGYIYNYKT....
tr|A0A6C1BRW5|A0A6C1BRW5_9AQUI  409  IAQ.EISSAVQE..VKGKKAYKAIHFFFSCPVALAYLFGVAFGH.YDKGYIYNYSS....
tr|A0A3M1C218|A0A3M1C218_9BACT  327  IAK.ECASAVQE..VKGKKHHRRLHFFFSCPVVIAYLFGVAFGH.FGSGVIYNFEG....
tr|A0A5P1V5W6|A0A5P1V5W6_9BACT  418  VAK.EAASFIQD..VREKYSFNSYHFFFSCPVVVAFMIGLAFGH.YVDGQIYNFQK....
tr|A0A1M6Q5V1|A0A1M6Q5V1_9AQUI  401  IAR.EIAGAIQE..VAKTATIKRFHFFFSCPVPIAFLVGVAFGH.YNPGSIYNYEQ....
tr|A0A7V3ZWQ1|A0A7V3ZWQ1_UNCW3  407  VAM.AISGIIQD..VKREHPFKCFHFFLSCPVPLAFLVGMAFGY.YSNGYIYNYQK....
tr|D3SNA5|D3SNA5_THEAH          405  IAR.QCASLLQE..IRTERDYKEFHFFFSSPVVFAFMLGVAFGH.YSPGYIYQYFD....
tr|A8UTR1|A8UTR1_9AQUI          405  VAK.ESASLIQD..LRSGKDYESFHFFLSVPVPVAFMVGVAFGC.YNPGYVYNYQG....
tr|W0DEP5|W0DEP5_9AQUI          403  VAT.ETASVIQN..IRREHSFEDFHFFLSTPVPIAFMGGLSFGH.YGEGYIYNYAG....
tr|A0A7X7TR99|A0A7X7TR99_9THEM  414  EVS.ELMSIVQK..VKAEKLLSKIEFFMSCPIPIAFGFGMAFGH.FGNGSIYAYNN....
tr|A0A841GNB0|A0A841GNB0_9BACT  412  IVS.EIMSVTQN..IQFKHSCENVFFFMSCPVPLSFGVGLSFGD.FAKGSIFHFDK....
tr|K2NM55|K2NM55_THEA7          296  IVS.EIMSVTQN..IQFKHSCENVFFFMSCPVPLAFGVGLSFGD.FAKGSIFHFDK....
tr|A0A7V4KD75|A0A7V4KD75_FERPE  427  IVS.ELFSITQI..VRNDFYYDELHIFLSCPVPIAFGFGMALGD.FVPGKIYNYFKGGKT
tr|A7HMU9|A7HMU9_FERNB          409  YVA.ELFTITQN..TKYILGYEEASYFLSIPVPIAFGLGMALGT.YKPGKIYHYDR....
tr|A0A101EV44|A0A101EV44_9THEM  404  YVC.ELFSVAQI..VKRKRAYRGASFFLSCPVPIAFGLGASFGD.YSPGFIYQYDQ....
tr|A0A432Q0P4|A0A432Q0P4_9AQUI  396  IVNEEIRS..........LEDKCYDLVLETSNDFAFALGYYLED.YKCLNLFHRGKFVYY
tr|A0A1V5WQ44|A0A1V5WQ44_9BACT  437  IVQ.ETNSLLNY..LKKSYHWQRLHIFQSSPNAICIALGIALGN.FTPATIYHYQPQA..
tr|B0VHB4|B0VHB4_CLOAI          436  YLQ.EINSALNT..ARQEYHWERIHLFQTAPTALCMALGIAVGH.FLPVDVYHYQFNA..
tr|A0A238XV58|A0A238XV58_9BACT  431  LVK.EIYNLLNCQTF...KKFSKLWFFMNLPVDIAFGLGSTFQD.TRMISLFKFFPG...
tr|C1DV83|C1DV83_SULAA          421  ISR.ELYTYINT......IKDK.IHLFYSIPVPISLSLGMAIAH.FKDITLYHYDS....
tr|B2V8L9|B2V8L9_SULSY          430  IVS.EIYTAIDD......NKQKENYMVFSAPVAIMLALGMALGY.FLPIKVFHYNR....
tr|C4FJQ9|C4FJQ9_9AQUI          289  IVS.EIYTAIDD......NKQKENYMVFSAPVAIMLALGMALGY.FLPIKVFHYNR....
tr|F8E961|F8E961_FLESM          431  TVA.EIYSLVDEAGELIGKSIRKYHFFMSIPVPVAFGFGMAYGD.YKKIAVYNYVK....
tr|E4TFD9|E4TFD9_CALNY          427  VVS.EIYSLLTENILDFKNVIKKYHFIFSVPVPIAFALGMAIGD.YKKILVYNFDK....
tr|A0A1R1MKQ3|A0A1R1MKQ3_9BACT  420  IVS.EIYSELNR..LRSEYMVERFHIFLSIPCPMAFALGACAGK.FIPATIYNYVP....
tr|A0A238Y624|A0A238Y624_9BACT  416  ISS.EIYSEINR..LRLKEKVKRFHIFLSVPCPIAFTVGAASGH.FIPATVYNYHF....
tr|A0A285NI51|A0A285NI51_9AQUI  438  IVS.EIYSVISV..LKYRYKIKRFHLFLSVPVPIATALGMALGH.FIDIIVYNYNTLPDA
tr|A0A432RBX1|A0A432RBX1_9AQUI  439  ITR.EIFTVIND..MKN.KKYERIHIFLSSPVAISFASGMAIGH.FINGTVYNINK.N..
tr|A0A2N7Q1Y2|A0A2N7Q1Y2_9AQUI  360  IVR.EIYSALNM..L.K.DRYKKLHIFLSVPVPIAFCLGMAVGH.FMNATIYNYNTKS..
```

```
tr|A0A4R1GDP6|A0A4R1GDP6_9BACT   446 PL.KDYRPVYNLGEVENV..........................................
tr|A0A1E3G168|A0A1E3G168_9BACT   483 GE...YRLVYSNIQKQ............................................
tr|A0A7C5U2L3|A0A7C5U2L3_9BACT   475 GD...YHLVFSNV...............................................
tr|A0A285P190|A0A285P190_9AQUI   462 N..ELYERVLSLEFLRDLIEKA......................................
tr|A0A6C1BRW5|A0A6C1BRW5_9AQUI   461 KD.ITYEQVLALEFLRSLREGGYIINMG.G..............................
tr|A0A3M1C218|A0A3M1C218_9BACT   379 ...NTYEPVLELEFLRGLIED.......................................
tr|A0A5P1V5W6|A0A5P1V5W6_9BACT   470 GE.NLYQPVIDFKFLRKIREKRSD....................................
tr|A0A1M6Q5V1|A0A1M6Q5V1_9AQUI   453 GT...YKKVLDIEFLRDLRES.......................................
tr|A0A7V3ZWQ1|A0A7V3ZWQ1_UNCW3   459 GQEEVYVKAVSLEKVRKIREN.......................................
tr|D3SNA5|D3SNA5_THEAH           457 GT...YVKVLDISHLKAIREGKNLALSG.ETKTP..........................
tr|A8UTR1|A8UTR1_9AQUI           457 KS...YAKVIGLESIRRIREEGGEVSP.................................
tr|W0DEP5|W0DEP5_9AQUI           455 GT...YEPVVSFSFLKALREGKYVLSEV................................
tr|A0A7X7TR99|A0A7X7TR99_9THEM   466 IE.NTYTAVIRIEELK............................................
tr|A0A841GNB0|A0A841GNB0_9BACT   464 NT.GDYIEVFKIEKIRGEVL........................................
tr|K2NM55|K2NM55_THEA7           348 NT.GDYIEVFKIEKIRG...........................................
tr|A0A7V4KD75|A0A7V4KD75_FERPE   483 KK.AGYIPVLDINEILR...........................................
tr|A7HMU9|A7HMU9_FERNB           461 ER.ADYFEVIDISKLSNR..........................................
tr|A0A101EV44|A0A101EV44_9THEM   456 NS.SSYVPVFQTDLIFQLVLSNS.....................................
tr|A0A432Q0P4|A0A432Q0P4_9AQUI   445 LQNPSNKPYFILDKCPLYLLNTRMFHLHVKEVSPEIFSSSVKKYGYVNLVEDQDILKVLN
tr|A0A1V5WQ44|A0A1V5WQ44_9BACT   491 .EGEHYKGMYNLEMVK...NYRPQYVPQT...............................
tr|B0VHB4|B0VHB4_CLOAI           490 .EEPKYRCVFSLDKML...NL.......................................
tr|A0A238XV58|A0A238XV58_9BACT   483 .KKDKYVEVLNVSPGPSPGGVKSIF...................................
tr|C1DV83|C1DV83_SULAA           468 .KNTTYIKIPINLNE.....VRSKF...................................
tr|B2V8L9|B2V8L9_SULSY           478 .DE..YIEVPIKLNEE...ILRSPF...................................
tr|C4FJQ9|C4FJQ9_9AQUI           337 .DE..YIEVPIKLNEE...ILRSPF...................................
tr|F8E961|F8E961_FLESM           485 .SQSTYKKVADSDLSR...NLKMAF...................................
tr|E4TFD9|E4TFD9_CALNY           481 .QIGSYIKVFDSDLKE...ELPMNAF..................................
tr|A0A1R1MKQ3|A0A1R1MKQ3_9BACT   472 .ERNSYSPVIKTD..K...LEN.IF...................................
tr|A0A238Y624|A0A238Y624_9BACT   468 .TEDIYKPVINTE..K...IEN.PF...................................
tr|A0A285NI51|A0A285NI51_9AQUI   494 LDKELYFPVFNLKDEI...LKS.KF...................................
tr|A0A432RBX1|A0A432RBX1_9AQUI   491 .DETPYFATFNIEDKA...LYS.MF...................................
tr|A0A2N7Q1Y2|A0A2N7Q1Y2_9AQUI   412 .QDTPYYPVFDTFDER...LKS.HF...................................
```

```
tr|A0A4R1GDP6|A0A4R1GDP6_9BACT       .......................................
tr|A0A1E3G168|A0A1E3G168_9BACT       .......................................
tr|A0A7C5U2L3|A0A7C5U2L3_9BACT       .......................................
tr|A0A285P190|A0A285P190_9AQUI       .......................................
tr|A0A6C1BRW5|A0A6C1BRW5_9AQUI       .......................................
tr|A0A3M1C218|A0A3M1C218_9BACT       .......................................
tr|A0A5P1V5W6|A0A5P1V5W6_9BACT       .......................................
tr|A0A1M6Q5V1|A0A1M6Q5V1_9AQUI       .......................................
tr|A0A7V3ZWQ1|A0A7V3ZWQ1_UNCW3       .......................................
tr|D3SNA5|D3SNA5_THEAH               .......................................
tr|A8UTR1|A8UTR1_9AQUI               .......................................
tr|W0DEP5|W0DEP5_9AQUI               .......................................
tr|A0A7X7TR99|A0A7X7TR99_9THEM       .......................................
tr|A0A841GNB0|A0A841GNB0_9BACT       .......................................
tr|K2NM55|K2NM55_THEA7               .......................................
tr|A0A7V4KD75|A0A7V4KD75_FERPE       .......................................
tr|A7HMU9|A7HMU9_FERNB               .......................................
tr|A0A101EV44|A0A101EV44_9THEM       .......................................
tr|A0A432Q0P4|A0A432Q0P4_9AQUI   505 ERFQIWSEHKNWATLNRGDRVFLIRREKGRLRFFEILLT
tr|A0A1V5WQ44|A0A1V5WQ44_9BACT       .......................................
tr|B0VHB4|B0VHB4_CLOAI               .......................................
tr|A0A238XV58|A0A238XV58_9BACT       .......................................
tr|C1DV83|C1DV83_SULAA               .......................................
tr|B2V8L9|B2V8L9_SULSY               .......................................
tr|C4FJQ9|C4FJQ9_9AQUI               .......................................
tr|F8E961|F8E961_FLESM               .......................................
tr|E4TFD9|E4TFD9_CALNY               .......................................
tr|A0A1R1MKQ3|A0A1R1MKQ3_9BACT       .......................................
tr|A0A238Y624|A0A238Y624_9BACT       .......................................
tr|A0A285NI51|A0A285NI51_9AQUI       .......................................
tr|A0A432RBX1|A0A432RBX1_9AQUI       .......................................
tr|A0A2N7Q1Y2|A0A2N7Q1Y2_9AQUI       .......................................
```
